# Bioengineered MSC^*Cxcr2*^ transdifferentiated keratinocyte-like cell-derived organoid potentiates skin regeneration through ERK1/2 and STAT3 signaling in diabetic wound

**DOI:** 10.1007/s00018-023-05057-3

**Published:** 2024-04-10

**Authors:** Subholakshmi Choudhury, Neha R. Dhoke, Shilpa Chawla, Amitava Das

**Affiliations:** 1 Department of Applied Biology, Council of Scientific & Industrial Research-Indian Institute of Chemical Technology (CSIR-IICT), Uppal Road, Tarnaka, Hyderabad, 500007 TS India; 2 Academy of Scientific and Innovative Research (AcSIR), Ghaziabad, 201002 India

**Keywords:** Chronic non-healing wounds, Bioengineered MSCs, Keratinocytes, Cell transplantation, 3D skin organoids, Organoid grafting, Tissue regeneration

## Abstract

Skin regeneration is severely compromised in diabetic foot ulcers. Allogeneic mesenchymal stem cell (MSC) transplantation is limited due to the poor engraftment, mitogenic, and differentiation potential in the harsh wound microenvironment. Thus, to improve the efficacy of cell therapy, the chemokine receptor *Cxcr2* was overexpressed in MSCs (MSC^*Cxcr2*^). CXCL2/CXCR2 axis induction led to the enhanced proliferation of MSCs through the activation of STAT3 and ERK1/2 signaling. Transcriptional upregulation of FGFR2IIIb (KGF Receptor) promoter by the activated STAT3 and ERK1/2 suggested trans-differentiation of MSCs into keratinocytes. These stable MSC^*Cxcr2*^ in 2D and 3D (spheroid) cell cultures efficiently transdifferentiated into keratinocyte-like cells (KLCs). An in vivo therapeutic potential of MSC^*Cxcr2*^ transplantation and its keratinocyte-specific cell fate was observed by accelerated skin tissue regeneration in an excisional splinting wound healing murine model of streptozotocin-induced type 1 diabetes. Finally, 3D skin organoids generated using MSC^*Cxcr2*^-derived KLCs upon grafting in a relatively avascular and non-healing wounds of type 2 diabetic *db/db* transgenic old mice resulted in a significant enhancement in the rate of wound closure by increased epithelialization (epidermal layer) and endothelialization (dermal layer). Our findings emphasize the therapeutic role of the CXCL2/CXCR2 axis in inducing trans-differentiation of the MSCs toward KLCs through the activation of ERK1/2 and STAT3 signaling and enhanced skin regeneration potential of 3D organoids grafting in chronic diabetic wounds.

**Supplementary Information:**

The online version contains supplementary material available at 10.1007/s00018-023-05057-3.

## Introduction

Cutaneous wound healing is a complex dynamic process involving four overlapping stages—hemostasis, inflammation, cellular proliferation, and remodeling [1]. These processes result in a synchronized network of repair mechanisms like cell migration, proliferation, and tissue regeneration orchestrated by an interplay of cytokines, growth factors, extracellular matrix, and chemokines. The wound healing processes are severely impaired in patients with metabolic diseases like diabetes [2] and pathophysiological skin conditions like atopic dermatitis [3, 4], and psoriasis [5]. The resident cells within the wound microenvironment, such as fibroblasts, keratinocytes, and endothelial cells, are functionally compromised in patients with diabetic foot ulcers (DFU) due to persistent hyperglycemia that delays the healing process resulting in chronic non-healing wounds [1]. DFU is a major complication associated with diabetes mellitus. Approximately 19–34% of diabetic patients worldwide are at risk of developing DFU in their lifetime [6]. The process of wound healing in diabetic patients remains incomplete due to the downregulation of cytokines and growth factors, such as platelet-derived growth factor (PDGF), vascular endothelial growth factor (VEGF), keratinocyte growth factor (KGF), epidermal growth factor (EGF), transforming growth factor beta 1 (TGFβ1), insulin growth factor 1 (IGF-1), interleukin 6, and TNF-α [7]. The physiological functions of keratinocytes are impaired due to hyperglycemia and increased ROS, advanced glycation end products (AGEs), proinflammatory cytokines, and mitochondrial dysfunction, resulting in delayed wound healing [8]. Recent studies have demonstrated the crucial role of chemokines, which are a family of heparin-binding cytokines typically mediating directional migration and leukocyte trafficking, and their cognate receptors substantiate the recruitment of leukocyte subtypes during wound healing [9–11]. Functionally chemokines may be classified as pro-inflammatory, which are expressed by leukocytes and other cells only upon activation, and homeostatic, which are constitutively expressed. Thus, chemokines receptors can serve as useful bait to recruit the target cells for modulating the physiological processes in chronic wounds [12].

Mesenchymal stem cells (MSCs) are promising candidates for cell therapies for skin tissue regeneration in chronic wounds due to their ability to transdifferentiate into multiple cell lineages and low immunogenicity. Though the therapeutic potential of MSCs has been demonstrated in several preclinical studies, particularly in cutaneous regeneration [13], MSC transplantation has demonstrated limited success to enhance wound healing in clinical settings of chronic diabetic wounds [14, 15]. A major drawback of MSC transplantation is due to its low engraftment, survival rate, and retort time to transdifferentiate in different lineages, which limits its clinical application. MSCs potentiate the repair and regeneration of injured tissues by modulating chemokine ligand–receptor interactions. Prior studies from our lab have demonstrated a higher expression of the chemokine ligand CXCL2 in the wound bed during the healing period [16]. This incited us to evaluate the effect of *Cxcr2* (the cognate receptor of CXCL2) modulation in MSC physiology. We demonstrated the transcriptional regulation and molecular mechanism of *Cxcr2*-mediated enhanced MSC trans-differentiation into keratinocytes-like cells (KLCs) in vitro and the efficacy of *Cxcr2*-bioengineered MSCs transplantation therapy for skin regeneration in the excisional splinting wound healing model in type 1 diabetic mice. Furthermore, we generated 3D skin organoids using *Cxcr2*-bioengineered MSC,derived KLCs and fibroblasts to evaluate the efficacy of these 3D organoid grafting-mediated skin regeneration by increased epithelialization (epidermal layer—keratinocytes), and vascularity (dermal layer) in type 2 diabetic transgenic *db/db* mice.

## Materials and methods

### Cell culture studies

Mouse bone marrow-derived mesenchymal stem cells (MSCs) (Cyagen, Santa Clara, USA) were cultured in α-MEM medium (HiMedia, Mumbai, India) supplemented with 10% fetal bovine serum (FBS) and 1% penicillin–streptomycin (Gibco, Billings, USA). Cells were maintained at 5% CO2 at 37 °C in a humidified atmosphere [16].

### Gene cloning studies

Sub-cloning of Cxcr2 gene on lentiviral vector for stable overexpression and/or silencing: Cxcr2-MycDDK gene insert was sub-cloned from pCMV-Cxcr2-MycDDK plasmid vector into the TurboGFP region of the lentiviral vector pCW57-TurboGFP-2a-MCS as described previously [17]. Lentiviral particles were generated by co-transfecting HEK-293T cells with the packaging vectors psPAX2, and pMD2.G along with pCW57-Cxcr2MycDDK, pCW57-TurboGFP, piLenti-Scrambled shRNA-GFP, and/or piLenti-Cxcr2 shRNA-GFP (ABM, Richmond, CA) using Lipofectamine 3000 (ThermoFisher, Waltham, USA). MSCs were transduced with the lentiviral particles using polybrene (10 μg/ml) and doxycycline (5 μg/ml) for transgene induction and subsequently subjected to puromycin selection (5 μg/ml) as described earlier [16, 17]. Gene cloning protocols were performed with prior approval of the Institutional Biosafety Committee (Approval No. IICT/IBSC/03/2019).

*Generation of bioengineered MSCs with stable Cxcr2 overexpression and/or silencing*: MSCs were either transiently transfected with the plasmid vectors *pCMV-Cxcr2-Myc,dDK and*/or *pCMV-EGFP* (control) or separately transfected with the lentiviral construct *pCW57-Cxcr2-MycDDK-2a-MCS* and/or *pCW57-TurboGFP-2a-MCS* (control) and for the silencing of the *Cxcr2* gene by transfecting the MSCs with *piLenti-Cxcr2-shRNA-GFP* and/or *piLenti-Scr-shRNA-GFP* (control). These transfected MSCs were subjected to RNA and protein isolation post 48 h and 72 h, respectively of culture [16].

### Promoter–reporter studies

In silico* analysis:* FGFR2IIIb (KGF Receptor, KGFR) promoter up to 5 kb upstream to the transcription start site confirmed the presence of one STAT3-binding site (BS) and four proximal SP1-binding sites (BS). Seven promoter–reporter constructs bearing all the transcription factor-binding sites (TFBS) depicted as Wild type (WT) and with sequential deletion of binding sites (e.g., ΔBS1) were designed and cloned as described previously [16].

*Promoter cloning studies:* Two constructs of the FGFR2IIIb promoter were designed containing the *STAT3* BS (*FGFR2IIIb STAT3* WT—179 bp), and deletion construct without *STAT3* BS (*FGFR2IIIb* Δ *STAT3*—86 bp) was cloned from mouse skin DNA using the restriction enzymes *EcoRI* and *BamHI* into the vector pMCS-Green *Renilla* Luc plasmid vector (ThermoFisher, Waltham, USA) as described previously [17]. Similarly, five constructs of the FGFR2IIIb promoter were designed containing four proximal SP1-binding sites (SP1 BS1-4), (*FGFR2IIIb ERK WT—*380 bp), three proximal SP1 BS (*FGFR2IIIb ERK ΔBS4—*140 bp), two proximal SP1 BS (*FGFR2IIIb ERK ΔBS4* + *3*—103 bp), one proximal SP1 BS (*FGFR2IIIb ERK ΔBS4* + *3* + *2*—91 bp), and no SP1 BS (*FGFR2IIIb ERK ΔBS4* + *3* + *2* + *1*—62 bp) and were cloned from mouse skin DNA using the restriction enzymes *EcoR1* and *BamH1* into the vector pMCS-Green *Renilla* Luc plasmid vector as described previously [16, 17].

*Promoter–Reporter dual luciferase assay: Cxcr2-*bioengineered MSCs with stable overexpression (MSC^*Cxcr2*^) and/or silencing (MSC^*Cxcr2 KD*^) along with their respective controls (MSC^*GFP*^ and MSC^*Scr*KD^) were plated in a 96-well plate at a cell density of 10^4^ cells/well and incubated for 24h at 37 °C. These *Cxcr2-*bioengineered MSCs were transfected with pMCS-Green *Renilla* Luc vector subcloned with the desired promoter region of mouse *FGFR2IIIb* (*KGFR*) and internal control vector (pCMV-Red *Firefly* Luc) using Lipofectamine 3000 (Invitrogen, USA). 24h post-transfection, these *Cxcr2*-bioengineered MSCs were treated with CXCL2 in absence or presence of pharmacological inhibitors of CXCR2–SB265610, ERK—PD98059, and STAT3—S3I201 at 37 °C for 72h. Promoter–reporter assays were performed according to the manufacturer's protocol [17].

### Gene and Protein expression studies

*Quantitative-Polymerase Chain Reaction (qPCR) analysis:* Total RNA was extracted from the bioengineered MSCs with stable *Cxcr2* overexpression and/or silencing followed by cDNA synthesis. The cDNA was subjected to qPCR analysis using mouse gene-specific primers (Table ST1) to calculate the relative fold change of various epithelial and keratinocyte lineage-specific genes, along with the chemokine receptor, *Cxcr2* [17, 18].

*Immunoblot analysis:* Cellular protein was extracted from *Cxcr2-*bioengineered MSCs (with stable overexpression and/or silencing of CXCR2) and their respective controls with RIPA lysis buffer and subjected to 10% SDS-PAGE. The protein was then transferred to a polyvinylidene difluoride membrane. The membrane was incubated with primary antibodies against mouse-specific CXCR2, FLAG-Tag, and Myc-Tag, along β-Tubulin, and β-Actin as loading controls. Separately, *Cxcr2*-bioengineered MSCs with stable overexpression and/or silencing were treated with CXCL2, in the absence/presence of CXCR2 inhibitor, SB265620, and various signaling pathway inhibitors. The cellular protein was extracted after 48 h of treatment and subjected to SDS-PAGE as described earlier [19], and the membrane was incubated with primary antibodies against the signaling mediators p-Src, t-Src, p-Akt, t-Akt, p-ERK1/2, t-ERK1/2, p-STAT3, and t-STAT3.

### Physiological assays

#### Proliferation assays

The proliferative potential of the bioengineered MSCs was evaluated using BrdU incorporation, colony formation, and MTT assays as described previously [16, 17]. Briefly, MSCs at 5 × 10^3^ cells per well were seeded in a 96-well plate and incubated with/without transfection of transgenes for 48 h followed by treatment with increasing concentration of CXCL2 (BioLegend, San Diego, USA) (0–100 ng/ml) in absence or presence of CXCR2 inhibitor, SB265610 (Merck, Darmstadt, Germany) (0–100 µM) along with their respective controls as described previously [16].

*BrdU incorporation assay:* Post-treatment, MSCs were added with BrdU solutions (Roche, Basel, Switzerland) as mentioned in the manufacturer’s protocol. MSCs were fixed, permeabilized, and incubated with the anti-BrdU-Peroxidase antibody for 1h and the detection signal was developed by adding its substrate followed by measurement of absorbance at 370 nm with a reference wavelength at 490 nm as described previously [17].

*Colony-Formation assay:* The control, as well as transfected MSCs, was seeded at a density of 100 cells/well in a 6-well plate and incubated for 14 days along with different treatments followed by fixation and staining as described earlier [18]. The number of colonies displaying 5 or more cells was scored under an inverted microscope.

*MTT assay:* MSCs with stable overexpression and/or silencing of *Cxcr2* were treated with CXCL2 (30 ng/ml), in the absence or presence of SB265620 (10 μM), and/or signaling pathway inhibitors PP2 (20 μM), Wortmannin (10 μM), PD98059 (30 μM), and S3I-201 (10 μM). After 48 h of incubation, the MTT assay was performed as described previously [18]. Separately, MSC,derived KLCs were treated with KGF (BioLegend, San Diego, USA) (10 ng/ml) in the absence or presence of Wortmannin (10 μM), and FAK14 (20 μM).

### Migration assays

*Boyden Chamber assay:* Chemotaxis-mediated cell migration was evaluated using the Boyden chamber [17]. Briefly, *Cxcr2-*bioengineered MSCs (overexpressing *Cxcr2*) were plated at a density of 1×10^3^ cells/well in the upper chamber Boyden chamber assembly and CXCL2 in the lower chamber as a chemoattractant. Separately, these MSCs were treated with CXCR2 inhibitor, SB265610 in the upper chamber as described previously [16].

### Differentiation assays

Transdifferentiation of Cxcr2-bioengineered MSCs into keratinocyte-like cells (KLCs): *Cxcr2-bioengineered MSC trans-differentiation in 2D culture: Cxcr2*-bioengineered MSCs with stable overexpression and/or silencing were cultured for 14 days in a keratinocyte expansion medium containing keratinocyte growth supplement (HiMedia, Mumbai, India) for trans-differentiation into keratinocyte-like cells (KLCs). Post-trans-differentiation, KLCs were subjected to RNA isolation and immuno-cytochemical analysis to evaluate the differential expression of keratinocyte and epithelial markers.

*Immunocytochemical analysis:* Transdifferentiated KLCs were fixed using 4% paraformaldehyde, permeabilized with Triton X-100 followed by blocking with 1% BSA for 1 h. Subsequently, the cells were incubated overnight with primary antibodies against keratinocyte markers, Involucrin (IVL) and Cytokeratin 5 (CK5), keratinocyte growth factor receptor FGFR2IIIb and phospho-tyrosine (p-Tyr) followed by 45-minute incubation with Alexa Fluor 488/555-conjugated secondary antibodies. DAPI containing mounting medium was used for counterstaining the nuclei [17]. The images were captured using confocal microscopy (Olympus FV10i, Olympus).

*Cxcr2-bioengineered MSC transdifferentiation in 3D culture:* The 3D spheroid culture was performed by plating 10^4^ *Cxcr2*-bioengineered MSCs with stable overexpression and/or silencing of *Cxcr2* per well, in low attachment round bottom 96-well plate (Corning, USA). The 96-well plate was coated with type 1 collagen solution (3 mg/mL, Merck, Germany) before MSC seeding. After 24h, the medium was changed into keratinocyte expansion media (Himedia, India) for trans-differentiation of bioengineered MSC spheroids toward keratinocyte lineage. The cells were cultured for 14 days, bright-field microscope images were captured on days 3, 7, and 14, and the spheroid area was measured to evaluate the spheroid forming efficiency [20]. As described above, bioengineered MSCs were fixed for immuno-cytochemical analysis with the primary antibodies against CK14, Myc tag, IVL, and CK5, and RNA was isolated in a separate set of experiments to evaluate the gene expression of keratinocyte markers.

### Generation of skin organoids

*Stratified epithelium formation using Cxcr2-bioengineered MSC,derived KLCs:* 3D skin organoids were generated in 24-well membrane inserts placed on a 24-well cell culture plate as mentioned previously [21]. NIH-3T3 cells were gently mixed in a 1:1 solution of type 1 collagen (3 mg/ml, Merck, Germany), and DMEM/F12 medium containing 5% FBS, 5 μg/mL insulin, and 10 ng/mL epidermal growth factor (EGF). The solution was transferred to the inserts and incubated for 30 min at room temperature. After confirming the gelation, 500 µL of the medium was added to the top of the insert and 1 mL to the bottom of the well. The matrix of fibroblasts and collagen was incubated at 37 °C with 5% CO_2_ for 7 days for the formation of a dermal equivalent. After 7 days, *Cxcr2*-bioengineered MSC,derived KLCs or control MSC^*GFP*^-derived KLCs were seeded onto each fibroblast layer and incubated at 37 °C with 5% CO2 for 30 min. 2 ml of low calcium epithelial medium (DMEM/F12 containing 4 mM L-glutamine, 10 μg/mL insulin, and 0.1% FBS) was added to the top of the insert and 3 mL to the bottom. Post 48 h of incubation, the medium was replaced with normal calcium medium (1.8 mM calcium chloride) and further incubated for 48 h. Subsequently, all the media were aspirated, and 3 mL of cornification medium (DMEM/F12 medium containing 4 mM L-glutamine, 10 μg/mL insulin, 2% FBS, and 1.8 mM calcium chloride) was added only to the bottom well to generate an air-liquid interface. The 3D skin organoid was maintained for 14 days at 37 °C with 5% CO2 and the medium was changed every other day. The 3D skin organoid was harvested by cutting the edge of the insert [21] and used for further gene and protein expression and organoid transplantation as skin graft studies. Keratinocyte-specific gene expression analysis was performed in the RNA isolated from the stratified skin organoids as described above. Separately, whole mounts of the generated 3D skin organoids were co-immunostained with Myc tag and CK5 antibodies, and separately with IVL and CK14 antibodies as described above.

### In vivo studies

*Generation of full excisional splinting wound healing model in Type 1 diabetes mice:* Type 1 diabetes was generated in 6–8 weeks old, male, or female *C57BL/6* mice by *intraperitoneal* administration of streptozotocin (STZ) at a dose of 70 mg/kg body weight for five consecutive days as described previously [16, 17]. Diabetes generation was confirmed by monitoring blood glucose levels at regular intervals for 2 weeks and intermittently till day 14 post-surgery. Excisional wounds were generated on the dorsal side using a 5 mm biopsy punch. Type 1 diabetic mice with two wounds on their dorsum were grouped separately and used for transplantation studies.

*Cxcr2-bioengineered MSC transplantation therapy for skin regeneration in type 1 diabetes: Cxcr2-*bioengineered MSCs with stable overexpression (MSC^*Cxcr2*^) and/or silencing (MSC^*Cxcr2 KD*^) along with their respective controls (MSC^*GFP*^ and MSC^*Scr*KD^) were transplanted *intradermally* onto the wound periphery (1 × 10^6^ cells/wound). Doxycycline (2 mg/ml) was administered orally in the drinking water to the mice. A silicone splint to prevent healing by contraction, Tegaderm to prevent infection by covering the wound with transparent film, and adhesive bandage were used for dressing the wounds as described previously [16, 17, 22]. Morphometric wound healing analysis was performed by imaging while monitoring the mice on the 3rd, 5th, 7th, 10th, and 14th day post-surgery. The animals were euthanized after 14 days, and skin tissues were collected for further analysis. Morphometric wound healing analysis and histological analysis (hematoxylin–eosin, Sirius red staining) were performed to determine the rate of wound closure and tissue regeneration as described previously [17]. In vivo, engraftment and the fate of MSCs were evaluated using qPCR analysis and immunohistochemical analysis of the skin-specific keratinocyte and epithelial markers along with CXCR2 and Myc/GFP tag.

*Immunohistochemical analysis:* The microscopic slides containing skin tissue sections were deparaffinised with Xylene for 30 min and incubated in a gradient of ethanol (100–30% followed by distilled water, 5 min each). The slides were then incubated in antigen retrieval solution (1X citrate buffer and 0.05% Tween 20) at 90 °C for 15 min followed by blocking with goat serum for 1 h. The skin sections were co-immuno-stained with the primary antibodies against CXCR2/Myc tag, IVL/CK14, GFP/CXCR2, GFP/CK14, IVL/CXCR2, and α-SMA/CD31. Fluorescence intensity was determined using ImageJ software (ImageJ, NIH), and co-localization was calculated using Pearson’s correlation coefficient.

*Skin organoid grafting on type 2 diabetic db/db transgenic mice:* Skin organoids generated from *Cxcr2*-bioengineered MSC (MSC^*Cxcr2*^)-derived KLCs or control MSC^*GFP*^-derived KLCs were transplanted onto the excisional splinting wounds in type 2 *db/db* transgenic male mice, 10–12 months old along with the controls (un-transplanted and collagen matrix without cells). Rate of wound closure, histological (hematoxylin–eosin and Sirius red staining), and immuno-histochemical (skin-specific keratinocyte, epithelial markers, and signaling mediators along with CXCR2 and Myc/GFP tag) analyses were performed to determine the skin tissue regeneration as mentioned previously [16, 17, 22].

### Statistical analysis

All the experiments were performed at least thrice, and the data were represented as mean ± standard deviation of the mean (SD). Statistical significance was evaluated using one-way or two-way ANOVA followed by appropriate analysis such as post hoc or Tukey’s multiple comparison test or Student’s paired/un-paired *t* test (using GraphPad Prism 9.5.1). In immunofluorescence analysis, the correlation between two variables was analyzed using Pearson’s correlation coefficient. A *P* value of less than 0.05 was considered significant. Representative immunoblot images and photomicrographs from the experiments were reproduced at least thrice.

## Results

### Activation of the CXCL2/CXCR2 axis enhanced the proliferation and colony-forming efficiency of MSCs

Chemokine ligand–receptor interactions not only mediate the recruitment of cells but also their physiology. Our prior findings on the temporal expression analysis of an array of chemokines at the mice wound bed revealed a significant increase in the expression of the chemokine ligand CXCL2 post-surgery during the early to late stages of wound healing along with a concomitant increase in the expression of its cognate receptor CXCR2 in mouse bone marrow-derived MSCs [16]. This incited us to evaluate the effect of the CXCL2/CXCR2 axis in MSC physiology. A concentration-dependent increase in the MSC proliferation was observed in the presence of CXCL2 with a significant increase at 30 ng/ml (Fig. S1a). The CXCL2-induced proliferation was significantly abrogated in the presence of a pharmacological inhibitor of CXCR2, SB265610 (10 µM) as observed using BrdU incorporation (Fig. S1b) and colony-forming units (Fig. S1c) analyses indicating a plausible role of the CXCL2/CXCR2 axis in inducing the mitogenicity of MSCs. However, CXCL2 did not induce any significant change in the migration of MSCs even at higher concentrations indicating the specificity of the CXCL2/CXCR2 axis in inducing the proliferation but not migration of the MSCs (Fig. S1d). To further confirm these observations, MSCs were transiently transfected with either pCMV-*EGFP* (control) or pCMV-*Cxcr2-MycDDK* expression vectors, and overexpression was confirmed at mRNA (Fig. S2a-left panel) and protein levels (Fig. S2a-right panel). *Cxcr2* overexpression significantly enhanced the colony-forming efficiency as compared to the control which was further potentiated in the presence of CXCL2 (30 ng/ml) (Fig. S2b). The CXCL2/CXCR2 axis-mediated increase in colony formation was significantly perturbed in the presence of SB265610 (10 µM) as well as other pathway blockers (Fig. S2b). Further, CXCL2 induced the proliferation of MSCs (BrdU incorporation) in both the control and *Cxcr2* overexpressed groups were markedly abrogated by SB265610 (10 µM) as well as other pathway blockers (Fig. S2c, d). These observations confirmed the crucial role of the CXCL2/CXCR2 axis in mediating MSC proliferation.

### CXCL2/CXCR2 axis induced MSC proliferation via the activation of STAT3/ERK1/2 signaling

**Fig. 1:**
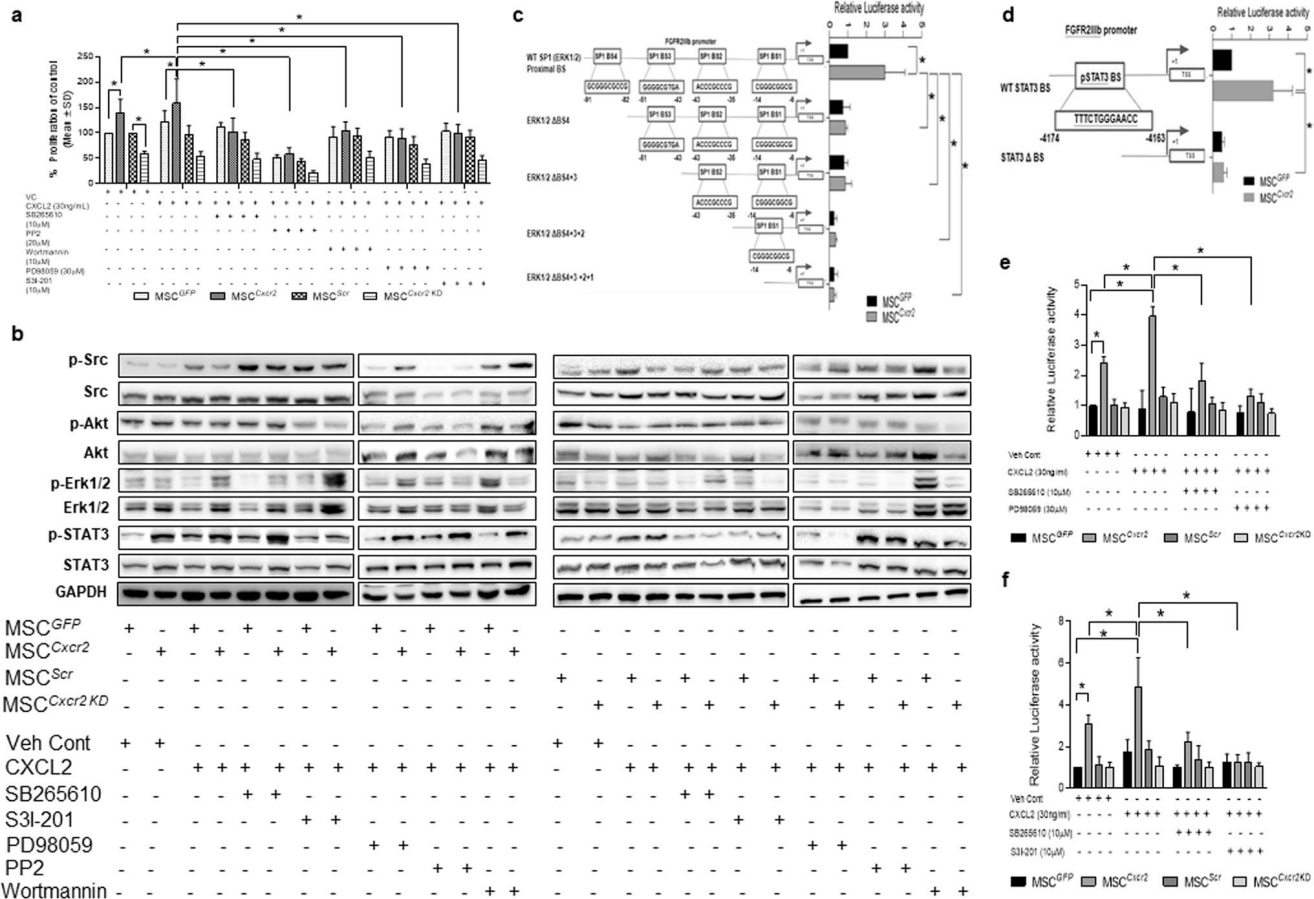
Molecular signaling of Cxcr2-mediated transcriptional regulation of FGFR2IIIb in MSCs **a** Graph depicting significantly increased proliferation of mouse bone marrow-derived MSCs with *Cxcr2* overexpression that was further increased with CXCL2, which was abrogated in the presence of CXCR2 inhibitor SB2656101 and signaling molecule inhibitors PP2 (Src), Wortmannin (Akt), PD98059 (ERK), and S31-201 (STAT3). **b** Immunoblot analysis showed increased expression of p-ERK1/2, and p-STAT3 with *Cxcr2* overexpression in MSCs which was reverted in the presence of the respective inhibitors, PD98059, and S3I-201. *Cxcr2* silencing in MSCs differentially downregulated the activation of these mediators. **c** In silico analysis of the *FGFR2IIIb* promoter showing the presence of four proximal SP1 (p-ERK1/2)-binding sites. Luciferase promoter–reporter assay with *Cxcr2*-bioengineered MSCs transfected with the promoter–reporter constructs revealed that SP1 BS4 is essential for *FGFR2IIIb* promoter activity. **d** Similarly, in silico analysis of the *FGFR2IIIb* promoter showing the presence of one STAT3-binding site and promoter–reporter assay depicting the positive role of STAT3 BS in *FGFR2IIIb* promoter activity. Graph representing CXCL2-mediated increase in the luciferase activity in **e** SP1 (ERK1/2) WT construct and **f** STAT3 WT construct of *FGFR2IIIb* promoter in MSC^*Cxcr2*^ which was abrogated by SB265610 and PD98059. (*n* = 3, *p < 0.05 as compared with MSC^*GFP*^, MSC^*Cxcr2*^ + CXCL2 in proliferation assay; *FGFR2IIIb SP1 (ERK1/2) WT/FGFR2IIIb STAT3 WT* construct in MSC^*GFP*^, *FGFR2IIIb SP1 (ERK1/2) WT*/*FGFR2IIIb STAT3 WT* construct in MSC^*Cxcr2*^; MSC^*GFP*^ Vehicle Control, MSC^*Cxcr2*^, and MSC^*Cxcr2*^ + CXCL2 in Luciferase-reporter assay)

To further evaluate the role of *Cxcr2* in modulating MSC physiology, *Cxcr2* was sub-cloned into a lentiviral vector to generate stable MSCs with *Cxcr2* overexpression (MSC^*Cxcr2*^) as described in Materials and Methods. MSCs were transduced with *Cxcr2* overexpressing and/or silencing lentiviral particles followed by puromycin selection. Overexpression of *Cxcr2* was confirmed by qPCR analysis (Fig. S3a) and immunoblotting with the tags FLAG and Myc, and CXCR2 (Fig. S3b). Similarly, the stable silencing of *Cxcr2* in MSCs (*MSC*^*Cxcr2 KD*^) *was* confirmed using qPCR analysis (Fig. S3c) and immunoblot analysis (Fig. S3d). MSC^*Cxcr2*^ depicted a significant increase in the percent proliferation which was further increased in the presence of CXCL2 (Fig. S3e). MSC^*Cxcr2 KD*^ showed a significant decrease in the percent proliferation as compared to the control MSC^*Scr*^ (Fig. S3e). The CXCL2-mediated increase in proliferation in MSC^*Cxcr2*^ was reverted in the presence of the CXCR2 inhibitor, SB265610, thereby confirming the role of *Cxcr2* in regulating MSC proliferation (Fig. S3e). Further, stable *Cxcr2* overexpression did not alter the migratory potential of MSCs (Fig. S3f). Next, to evaluate the molecular signaling pathway of *Cxcr2*-mediated increased MSC proliferation, MSCs with stable *Cxcr2* overexpression and/or silencing along with their respective controls were treated with CXCL2 in the absence or presence of signaling pathway inhibitors and subjected to MTT proliferation assay. A significant increase in the percent proliferation was observed in the MSC^*Cxcr2*^ group, which was reverted significantly in the presence of PP2 (a non-selective pan inhibitor of Src-family kinases), Wortmannin (a PI3K/AKT pathway inhibitor), PD98059 (ERK inhibitor), and S3I-201 (STAT3 inhibitor) (Fig. 1a). To confirm the specific signal transduction pathway of *Cxcr2*-mediated proliferation, immunoblot analysis was performed (Fig. 1b). Activation of ERK1/2 (phospho-ERK1/2) was observed in MSC^*Cxcr2*^ in the presence of CXCL2 (30 ng/ml) (Figure 1b, left panel), which was abrogated by CXCR2 inhibitor, SB265610 (10 µM). Further, with *Cxcr2* silencing a decreased expression of pERK1/2 was observed in the vehicle control group as well as in the presence of ERK inhibitor, PD98059 (30 µM) (Fig. 1b, right panel). Similarly, *Cxcr2* overexpression also led to STAT3 activation, which was downregulated by STAT3 inhibitor, S3I-201 (30 µM) (Fig. 1b, left panel). Interestingly, MSCs with stable *Cxcr2* silencing perturbed STAT3 activation in the presence of SB265610. (Fig. 1b, right panel). However, these MSCs with stable *Cxcr2* modulation did not alter the Src and AKT levels. These observations suggested a plausible role of ERK1/2 and STAT3 signaling in the *Cxcr2*-mediated downstream targets in MSCs.

### Activated STAT3 and ERK1/2 transcriptionally regulate the promoter activity of FGFR2IIIb in MSC^Cxcr2^

Activated STAT3 and ERK1/2 [16] are known to translocate into the nucleus and act as transcriptional co-activators of target genes. Activated ERK1/2 in turn activates by phosphorylating SP1 and binding to the SP1 transcription factor-binding site (TFBS) on its target promoter [16]. MSCs possess the plasticity property that enables them to be transdifferentiated into non-obvious lineages like Keratinocytes [22], which are the major cell type present in the epidermal layer of the skin. Keratinocytes express the receptor FGFR2IIIb, also known as Keratinocyte Growth Factor Receptor (KGFR) which binds to its cognate ligand FGF7 or KGF. The FGF7/FGFR2IIIb signaling regulates the proliferation, migration, and differentiation of skin keratinocytes [23]. The in silico analysis of the promoter sequence of mouse *FGFR2IIIb* (*KGFR*) revealed the presence of four putative proximal SP1 (p-ERK1/2) binding sites (−6 to −91) (Fig. 1c) and one putative p-STAT3-binding site (−4163 to −4174) (Fig. 1d). Wild type (*FGFR2IIIb SP1 (ERK1/2) WT*, and *FGFR2IIIb STAT3 WT*), and deletion mutant constructs (*ERK1/2 ΔBS4*, *ERK1/2 ΔBS4*+*3*, *ERK1/2 ΔBS4*+*3*+*2, ERK1/2 ΔBS4*+*3*+*2*+*1, and STAT3 ΔBS*) of *FGFR2IIIb* luciferase reporter constructs were cloned as described in the Materials and Methods. MSC^*Cxcr2*^ significantly induced the luciferase activity of the *FGFR2IIIb SP1 (ERK1/2) WT* (Fig. 1c), and *FGFR2IIIb STAT3* WT (Fig. 1d) reporter constructs as compared with respective control. Further, deletion of the fourth SP1-binding sites (*ERK1/2 ΔBS4)* significantly reduced the luciferase activity (Fig. 1c). However, subsequent sequential deletion of the remaining three SP1-binding sites (*ERK1/2 ΔBS3, ERK1/2 ΔBS2*, *ERK1/2 ΔBS1)* did not alter the promoter activity to any further extent suggesting that BS4 is the essential site for p-ERK1/2-mediated SP1 binding on *FGFR2IIIb* promoter (Fig. 1c). Similarly, deletion of the p-STAT3-binding site (*FGFR2IIIb STAT3* Δ*BS*) significantly reduced the promoter activity suggesting the role of STAT3 in regulating *FGFR2IIIb* (*KGFR*) transcription (Fig. 1d). Further, CXCL2 could significantly induce the activity of the WT ERK1/2 (Fig. 1e) and WT STAT3 (Fig. 1f) promoter in MSC^*Cxcr2*^ but not in the control, MSC^*GFP*^, and MSC^*Scr*^ and the negative control, MSC^*Cxcr2 KD*^ groups. Additionally, the presence of CXCR2 inhibitor—SB265610, ERK1/2 inhibitor—PD98059, and STAT3 inhibitor—S3I-201 significantly reverted the CXCL2-mediated increase in the luciferase activity (Fig. 1e, f) in MSC^*Cxcr2*^ suggesting a crucial role of activated ERK1/2 and STAT3 in MSC with stable *Cxcr2* overexpression-mediated transcriptional regulation of *FGFR2IIIb* (*KGFR*). One limitation of the present study is that the luciferase promoter–reporter assay was performed by segregating the FGFR2IIIb promoter into two parts, one with only the SP1-binding sites without the STAT3-binding site, and the other with only the STAT3-binding site without the SP1-binding sites. Incorporating both SP1- and STAT3-binding sites together can provide a better understanding of the impact of sequential deletion in the context of the full promoter of FGFR2IIIb. Overall, these data indicate that *Cxcr2* overexpression can potentiate the trans-differentiation of MSCs toward keratinocyte lineage.

### Cxcr2 overexpression enhanced the trans-differentiation of MSCs toward keratinocyte-like cells

**Fig. 2:**
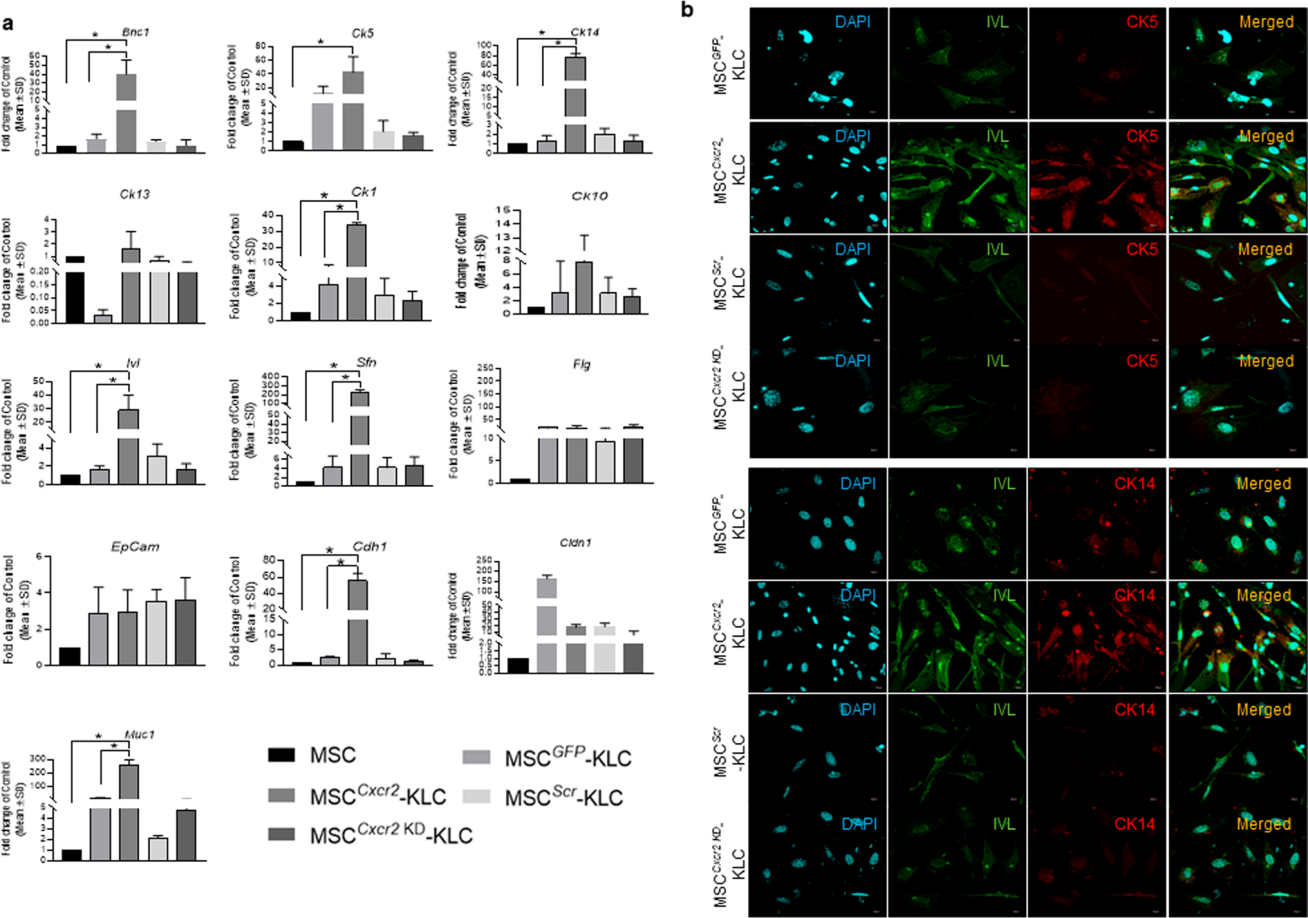
Cxcr2-mediated increased transdifferentiation potential of MSCs toward keratinocyte-like cells **a** Differential expression analysis of keratinocyte and epithelial markers in MSC^*GFP*^, MSC^*Cxcr2*^, MSC^*Scr*^, and MSC^*Cxcr2 KD*^-derived KLCs revealed a significant increase in the expression of Basonuclin (*Bnc1*), Cytokeratin 5, 14, 1, Involucrin (*Ivl*), Stratifin (*Sfn*), E-Cadherin (*Cdh1*), and Mucin-1 (*Muc1*) in MSC^*Cxcr2*^-KLCs. **b** Confocal microscopy images of MSC^*GFP*^, MSC^*Cxcr2*^, MSC^*Scr*^, and MSC^*Cxcr2 KD*^-derived KLCs immuno-stained with Involucrin and CK5 and **c** Involucrin and CK14 showing a marked increase in the expression of Involucrin, CK5, and CK14 in MSC^*Cxcr2*^-derived KLCs. (*n* = 3, *p < 0.05 as compared with MSC and MSC^*GFP*^-KLC)

Next, we wanted to evaluate the role of *Cxcr2* modulation in mediating the transdifferentiation of MSCs toward keratinocyte lineage. For transdifferentiation in 2D cell culture systems, *Cxcr2*-modulated MSCs and control MSCs were cultured in a complete keratinocyte expansion medium for 14 days as mentioned in the Materials and Methods. The expression of the keratinocyte markers (for primers refer to Table ST1) Basonuclin (*Bnc1*), cytokeratin 5 (*Ck5*), *Ck14*, *Ck1*, Involucrin (*Ivl*), and Stratifin (*Sfn*) was significantly increased in MSC^*Cxcr2*^-keratinocyte-like cells (KLC) as compared with MSC^*GFP*^-KLCs when cultured in the keratinocyte growth-specific medium (Fig. 2a). Further, significantly enhanced expression of the epithelial markers E-Cadherin (*Cdh1*) and Mucin-1 (*Muc1*) in MSC^*Cxcr2*^ suggesting the trans-differentiation of mesenchymal phenotype MSCs into epithelial phenotype KLCs (Fig. 2a). MSCs with stable *Cxcr2 KD* led to decreased expression of both keratinocytes and epithelial-specific gene expressions (Fig. 2a). Next, we evaluated the keratinocyte-specific protein markers in the *Cxcr2-*bioengineered MSCs using immunofluorescence analysis. We observed a marked increase in the fluorescence intensity of the CK5, CK14, and IVL in the MSC^*Cxcr2*^-KLCs as compared with MSC^*GFP*^, MSC^*Scr*^, and MSC^*Cxcr2*KD^.KLCs (Fig. 2b). These observations indicate the positive role of *Cxcr2* in increasing the trans-differentiation potential of MSCs toward KLCs.

### KGF activates the cellular physiology of transdifferentiated MSC^Cxcr2^-KLCs

FGFR2IIIb is a receptor tyrosine kinase that gets activated upon KGF binding and mediates the proliferation, migration, and differentiation of epithelial cells during wound healing. Immunofluorescence analysis of these *Cxcr2-*modulated MSC,derived KLCs in the absence or presence of KGF (10 ng/ml) revealed a marked increase in the co-localization of FGFR2IIIb (KGFR) and p-Tyr in the MSC^*Cxcr2*^ group in the presence of KGF (Fig. S4a, arrow-head denoting co-localization) as compared with the control, MSC^*GFP*^-KLCs. Further, MSC^*Cxcr2 KD*^-KLCs did not show any significant change in the co-localization of FGFR2IIIb, and p-Tyr as compared to MSC^*Scr*^-KLCs. Additionally, the presence of KGF did not alter the Pearson’s correlation coefficient in the MSC^*Scr*^ and MSC^*Cxcr2*KD^-KLCs (Fig. S4a). This indicates the activation of FGFR2IIIb only in the MSC^*Cxcr2*^-KLCs that in turn may induce the physiological phenotype in these cells. Next, we performed proliferation and migration assays of KLCs treated with KGF (FGF7) in the absence or presence of pharmacological inhibitors of the signaling pathway. The percent proliferation of MSC^*Cxcr2*^ -KLCs increased significantly in the presence of KGF (10 ng/ml) that was reverted significantly in the presence of signaling pathway inhibitors of AKT (Wortmannin, 10 µM), but not FAK (FAK14, 20 µM) (Fig. S4b). Furthermore, we observed a KGF-induced significant increase in the number of cells migrated in the MSC^*GFP*^-KLC and MSC^*Cxcr2*^-KLC groups that were reverted significantly in the presence of both AKT and FAK inhibitors (Fig. S4c). Further, *Cxcr2* silencing did not alter the migration of the MSC^*GFP*^-KLC and MSC^*Cxcr2*^-KLC groups (Fig. S4c). These results confirm the keratinocyte-specific cellular phenotype of MSC^*Cxcr2*^-derived KLCs.

### Cxcr2 overexpression increased the 3D spheroid forming efficiency during the transdifferentiation of MSCs toward keratinocyte-like cells

**Fig. 3:**
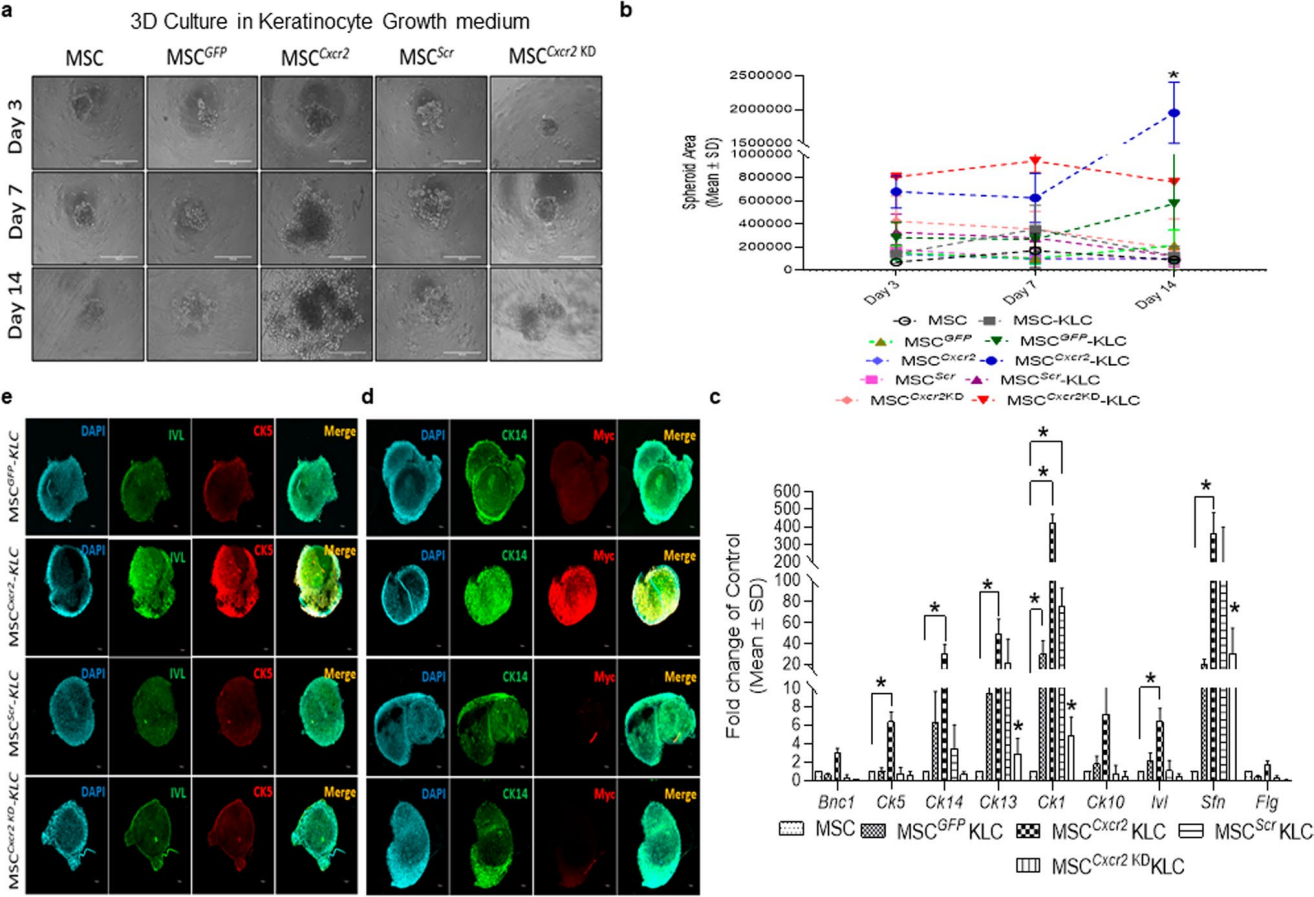
Enhanced spheroid forming efficiency of MSC^Cxcr2^-KLCs. **a** Representative bright-field microscopy images of *Cxcr2*-modulated MSCs in spheroid culture assay at days 3, 7, and 14. **b** Graph depicting a significant increase in MSC^*Cxcr2*^-KLC spheroid area (lower panel depicting the graph of linear regression analysis). **c** Differential expression analysis of keratinocyte markers in spheroids depicting a significant increase in the expression of *Ck5*, *Ck14*, *Ck13*, *Sfn*, and *Ivl*. Confocal microscopy images of spheroids immuno-stained with **d** CK14, and Myc, **e** Involucrin, and CK5, depicting increased expression in MSC^*Cxcr2*^-KLC spheroids. (*n* = 3, *p < 0.05 as compared with MSC-KLC/MSC^*GFP*^-KLC/MSC^*Scr*^-KLC/MSC^*Cxcr2*KD^-KLC; day 4/8 in spheroid area; and MSC in gene expression analysis)

One of the main properties of stem/progenitor cells is to self-organize into spheroid-like structures that can partially mimic the physiological functions of the tissue [20]. Subsequently, we wanted to evaluate the effect of *Cxcr2* modulation in MSCs undergoing keratinocyte trans-differentiation in 3D spheroid culture conditions. In the present study, stable *Cxcr2*-modulated MSCs were cultured in low adhesion plates to form spheroids as mentioned in the Materials and Methods. The size of the spheroids increased significantly from day 4 to day 14 in the MSC^*Cxcr2*^ cultured in keratinocyte expansion medium as compared with the controls (MSC^*GFP*^, MSC^*Scr*^) and negative control (MSC^*Cxcr2 KD*^) groups indicating enhanced spheroid forming efficiency of MSC^*Cxcr2*^ (Fig. 3a). Quantification of the 3D spheroids generated by MSC^*Cxcr2*^ groups cultured in keratinocyte expansion medium depicted significantly large-sized spheroids at day 14 as compared with their controls as well as those cultured in MSC expansion medium (Fig. 3b). Morphologically, the spheroids in the MSC^*Cxcr2*^ group demonstrated a proliferating layer of cells surrounding the core of the spheroid (Fig. 3a) correlating with the characteristics of epithelial spheroids which demonstrate a stromal core and an underlying surface-anchored keratinocyte layer [20]. Further, qPCR analysis of the keratinocyte and epithelial markers in the spheroids revealed a significant increase in the expression of the keratinocyte markers—*Ck5*, *Ck14*, *Ck13*, *Ck1*, *Ivl*, and *Sfn* (Fig. 3c). A significant decrease in the expression of *Ck13*, *Ck1*, and *Sfn* was observed with *Cxcr2* silencing. Immunofluorescence analysis depicted a marked increase in the expression of the keratinocyte markers CK14 along with Myc tag (Fig. 3d), and Involucrin, and CK5 (Fig. 3e) in MSC^*Cxcr2*^-KLC spheroids. These observations further confirm the trans-differentiation of MSCs toward KLCs and suggest the in vivo therapeutic potential of MSC^*Cxcr2*^ in enhancing tissue regeneration.

### Transplantation of MSC^Cxcr2^ accelerated wound closure and potentiated re-epithelialization at the wound bed in type 1 diabetic mouse model

**Fig. 4:**
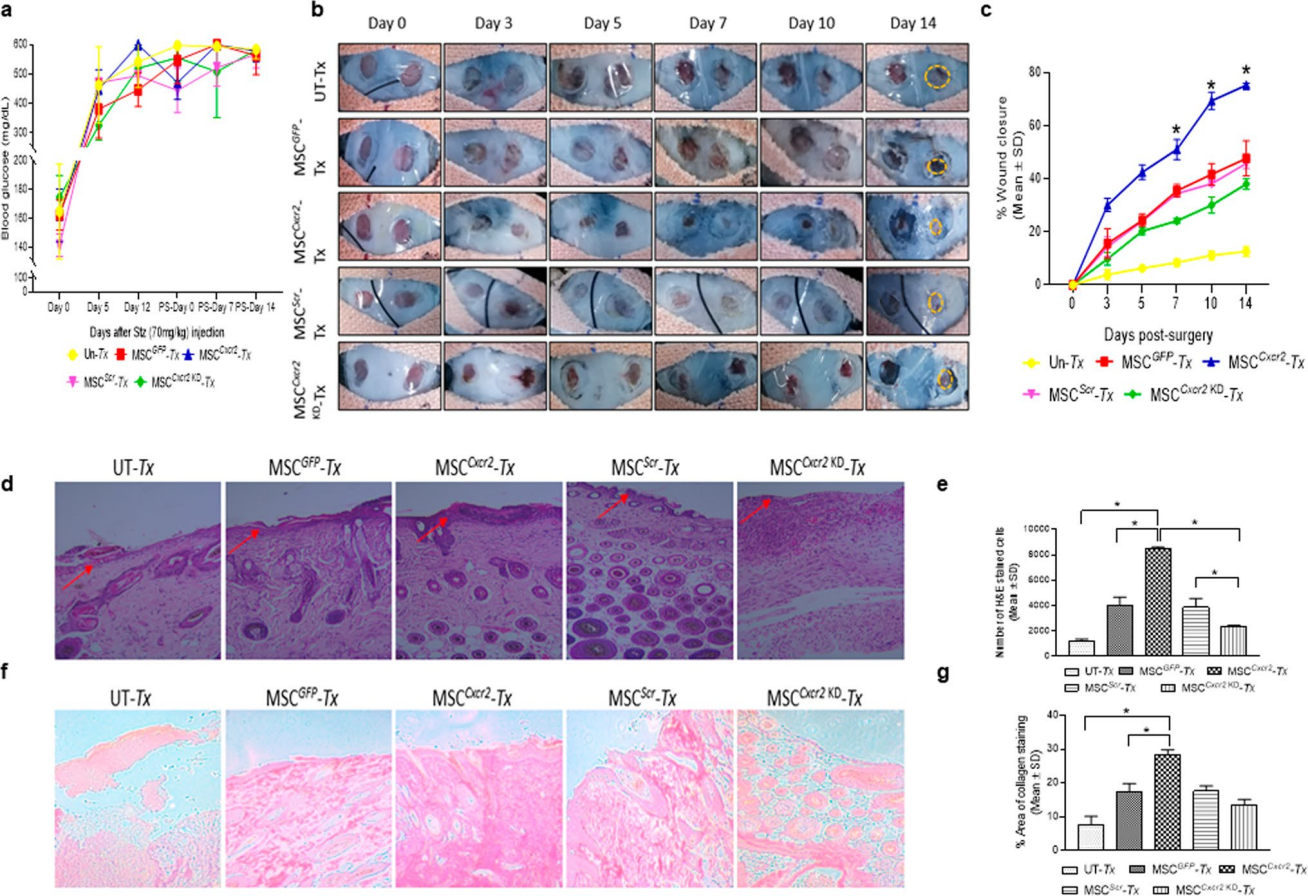
Accelerated wound closure with MSC^Cxcr2^ transplantation in type 1 diabetic mice **a** Graph depicting blood glucose levels in type 1 diabetic mice. **b** Representative images depicting morphometric analysis of type 1 diabetic wounds from post-surgery day 0 to day 14. **c** Graph depicting a significant increase in wound closure in the MSC^*Cxcr2*^* Tx* group from day 7 to day 14. **d** Representative images of hematoxylin and eosin (H&E) staining in regenerated type 1 diabetic mice wound tissue indicating epithelial thickness (marked by red arrow) and **e** quantitation of H&E-stained cells indicating increased granulation in the MSC^*Cxcr2*^ transplanted group. Similarly, representative images of **f** Sirius red staining and **g** Quantitative analysis depicting a significant increase in the percent area of collagen-stained tissue in the MSC^*Cxcr2*^* Tx* group, whereas no significant change was depicted in the control and negative control groups (UT-Untransplanted, *n* = 3, *p < 0.05 as compared with UT/MSC^*GFP*^/MSC^*Scr*^/MSC^*Cxcr2 KD*^ in morphometric analysis; and UT/MSC^*GFP*^/MSC^*Scr*^/MSC^*Cxcr2*^ in histology quantitation)

To evaluate the in vivo therapeutic potential of MSC^*Cxcr2*^, a full-thickness excisional splint wound healing model was generated in streptozotocin-induced type 1 diabetic mouse that was confirmed by the blood glucose levels (Fig. 4a). The type 1 diabetic mouse was transplanted *intradermally* at the wound periphery with 1 × 10^6^ cells/wound of MSC with stable *Cxcr2* overexpression and/or silencing groups as well as their respective controls. Morphometric wound healing analysis of the regenerated wound was performed which depicted markedly reduced wound size (Fig. 4b) as well as a significantly higher rate of wound closure (Fig. 4c) in the MSC^*Cxcr2*^ transplanted (*Tx*) group as compared with the MSC^*GFP*^ and/or MSC^*Scr*^ (controls) and MSC^*Cxcr2 KD*^ (negative control) *Tx* groups. This was further corroborated by hematoxylin–eosin (Fig. 4d, e) and Sirius red (Fig. 4f, g) staining for histological analysis of the regenerated wounds, which depicted the thickening of the epidermis layer (Fig 4d, S5), higher granulation tissue formation, and increased collagen deposition in the MSC^*Cxcr2*^ group (Fig. 4d-g). Also, the data suggest a higher number of H&E-stained infiltrated cells at the wound site of the MSC^*Cxcr2*^* Tx* group as compared with the MSC^*GFP*^ and/or MSC^*Scr*^ (controls), and MSC^*Cxcr2 KD*^ (negative control) *Tx* groups indicating *Cxcr2-*mediated accelerated wound tissue regeneration (Fig. S5)*.*

### MSC^*Cxcr2*^ transplantation increased the expression of keratinocyte and epithelial-specific markers at type 1 diabetic wound bed

**Fig. 5:**
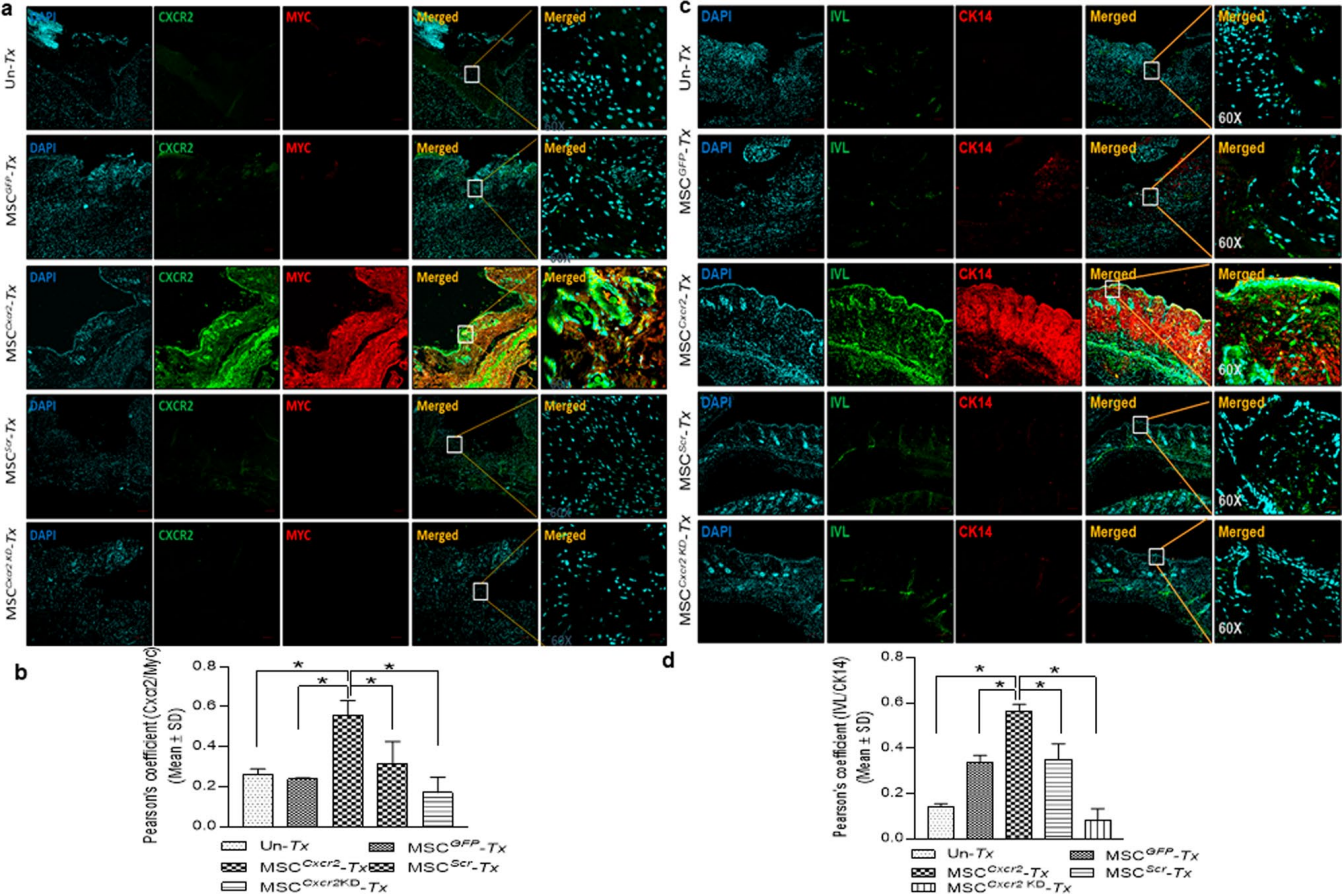
Enhanced re-epithelialization with MSC^Cxcr2^ transplantation at type 1 diabetic wound bed **a** Representative confocal microscopy images of regenerated type 1 diabetic mice wound tissue co-immunostained with CXCR2 and Myc tag, **b** showing significantly increased Pearson’s correlation coefficient in MSC^*Cxcr2*^* Tx* group. **c** Representative confocal microscopy images of type 1 diabetic mice wound tissue co-immunostained with the keratinocyte markers Involucrin and CK14, **d** showing significantly increased Pearson’s correlation coefficient in MSC^*Cxcr2*^* Tx* group (*n* = 3, *p < 0.05 as compared with Un-*Tx*/MSC^GFP^ *Tx*/MSC^*Scr*^* Tx*/MSC^*Cxcr2 KD*^* Tx*) (Scale bar: 10×–100 µm, 60×–20 µm)

For evaluation of the epithelial- and keratinocyte-specific gene expression, qPCR analysis was performed with the regenerated skin tissues post-surgery day 7 (Fig. S6a-c) and day 14 (Fig. S7a-c). The expression of the epithelial markers *Cdh1* and *Muc1* increased significantly in MSC^*Cxcr2*^ transplanted group as compared with the un-*Tx* and MSC^*GFP*^* Tx* group at day 7 (Fig S6b), whereas at day 14, a significant increase in the expression of the basal keratinocyte-specific markers *Bnc1*, *Ck5*, *Ck14*, intermediate markers *Ck13* and *Ck10*, and terminal differentiation markers *Ivl*, *Sfn*, and *Flg* was observed in the MSC^*Cxcr2*^ transplanted group as compared with the un-*Tx* group or differentially with the MSC^*GFP*^* Tx* group (Fig. S7a). The MSC^*Cxcr2*^* Tx* group also demonstrated a significant increase in the epithelial-specific markers *EpCAM*, *Cdh1*, Claudin (*Cldn1*), and *Muc1* as compared with un-*Tx* and MSC^*GFP*^* Tx* groups (Fig. S7b). Further, a significant increase in the MSC markers *CD73*, *CD90.2*, and *CD105* expression was observed on day 7 (Fig. S6c) but only *CD73* was significantly expressed at day 14 in the MSC^*Cxcr2*^* Tx* group (Fig. S7c) suggesting MSC^*Cxcr2*^-mediated accelerated wound tissue regeneration was occurring by accelerating the fate of the transplanted cells toward keratinocytes at the wound bed of type 1 diabetic mouse. For evaluation of the engraftment and fate of the transplanted cells, co-immunostaining of CXCR2 and Myc tag was performed which depicted a higher co-localization in the MSC^*Cxcr2*^* Tx* group as compared with the MSC^*GFP*^ or MSC^*Scr*^ (controls) and MSC^*Cxcr2 KD*^ (negative control) *Tx* groups (Fig. 5a) and quantification of co-localization (Fig. 5b). Since our control and negative control groups were GFP tagged while the MSC^*Cxcr2*^ lacks the GFP tag instead expressed Myc tag, we stained these sections with GFP and CXCR2. We observed an increased immunofluorescence intensity of GFP staining in the MSC^*GFP*^ or MSC^*Scr*^ (controls) and MSC^*Cxcr2 KD*^ (negative control) *Tx* groups while the MSC^*Cxcr2*^* Tx* group depicted higher immunofluorescence intensity of CXCR2 as compared with the others (Fig. S8a, b). Further, the co-immunostaining of the keratinocyte markers Involucrin (IVL) and CK14 also depicted higher co-localization in the MSC^*Cxcr2*^* Tx* group (Fig. 5c, d) suggesting enhanced re-epithelialization-mediated repair and regeneration of type 1 diabetic wound tissue. Similarly, immunostaining with GFP and CK14 revealed a higher immunofluorescence intensity of GFP in the control and negative control MSC *Tx* groups while the immunofluorescence intensity of CK14 was markedly higher in the MSC^*Cxcr2*^* Tx* group (Fig. S8c, d). Additionally, co-immunostaining of CXCR2 with the keratinocyte-specific marker Involucrin depicted higher co-localization in the MSC^*Cxcr2*^* Tx* group as compared with controls (Fig. S9a) and quantification of colocalization (Fig. S9b). Although the expression of α-SMA was found to be significantly higher in all the transplanted groups, the CD31 expression was not altered as compared to the un-transplanted group, indicating less blood vessel formation in the regenerated tissue (Fig. S9c,d). This incited us to generate 3D skin organoids containing the stratified epidermal and dermal layers.

### MSC^Cxcr2^-KLC,derived skin organoid grafting enhanced skin tissue regeneration in type 2 diabetic (db/db) transgenic mice wounds

**Fig. 6:**
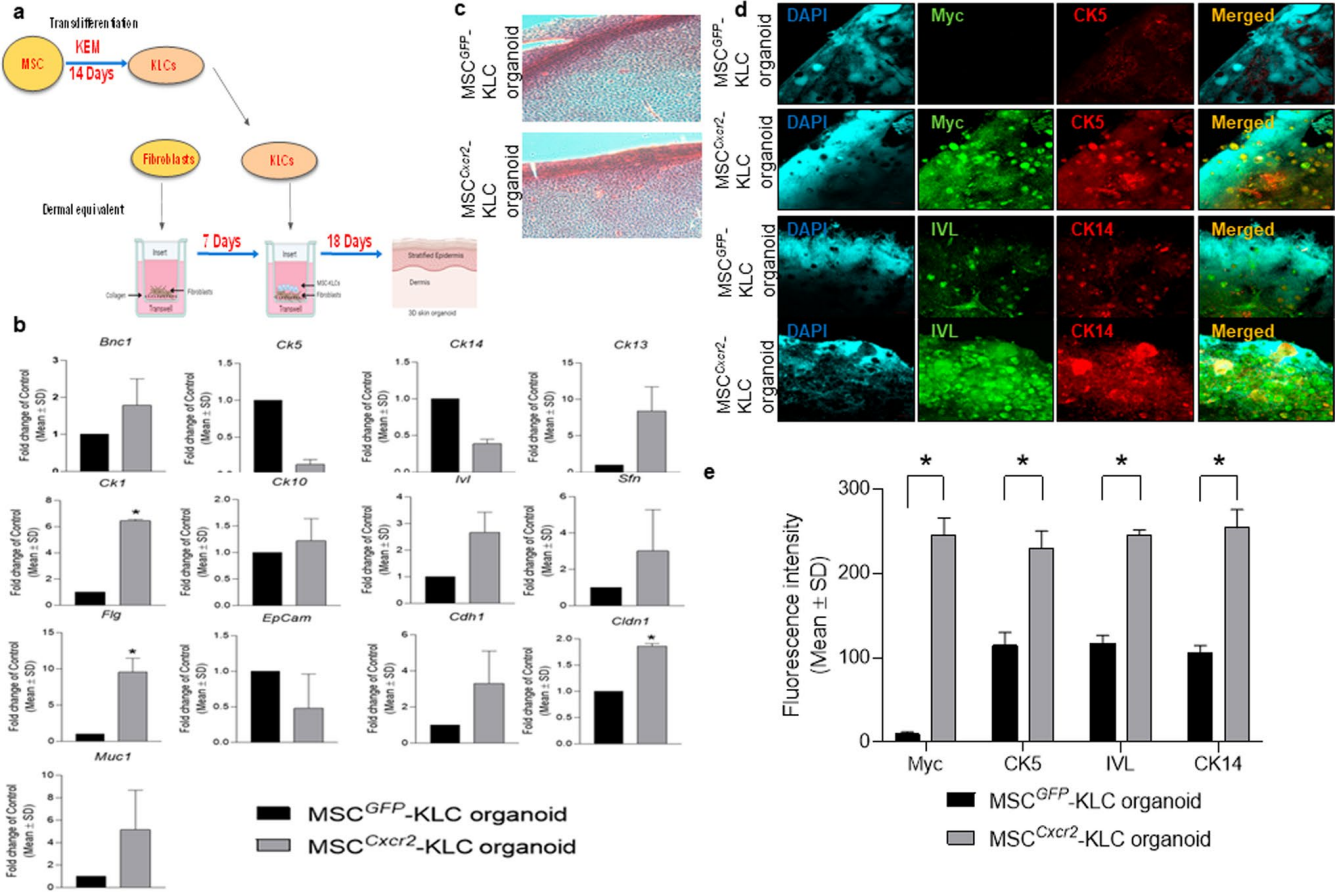
Generation of bi-layered 3D skin organoids with fibroblasts and bioengineered MSC-KLCs **a** Schematic representation of the organoid culture system. **b** Differential expression analysis of keratinocyte and epithelial markers in MSC^*GFP*^-KLC (control) organoids and MSC^*Cxcr2*^-KLC organoids depicting a significantly high expression of the keratinocyte markers *Ck1* and *Flg*, and epithelial marker *Cldn1* in MSC^*Cxcr2*^-KLC organoids. **c** Representative H&E-stained images of the skin organoids. **d** Representative confocal microscopy images of whole mount MSC^*GFP*^-KLC and/or MSC^*Cxcr2*^-KLC organoids, co-immunostained with Myc and CK5 (upper panel), and Involucrin and CK14 (lower panel). **e** Quantification of Pearson’s correlation coefficient depicting significant co-localization of Myc and CK5 (left), and Involucrin and CK14 (right) in MSC^*Cxcr2*^-KLC organoids (*n* = 3, *p < 0.05 as compared with MSC^*GFP*^-KLC organoids) (Scale bar: 60×–20 µm)

**Fig. 7:**
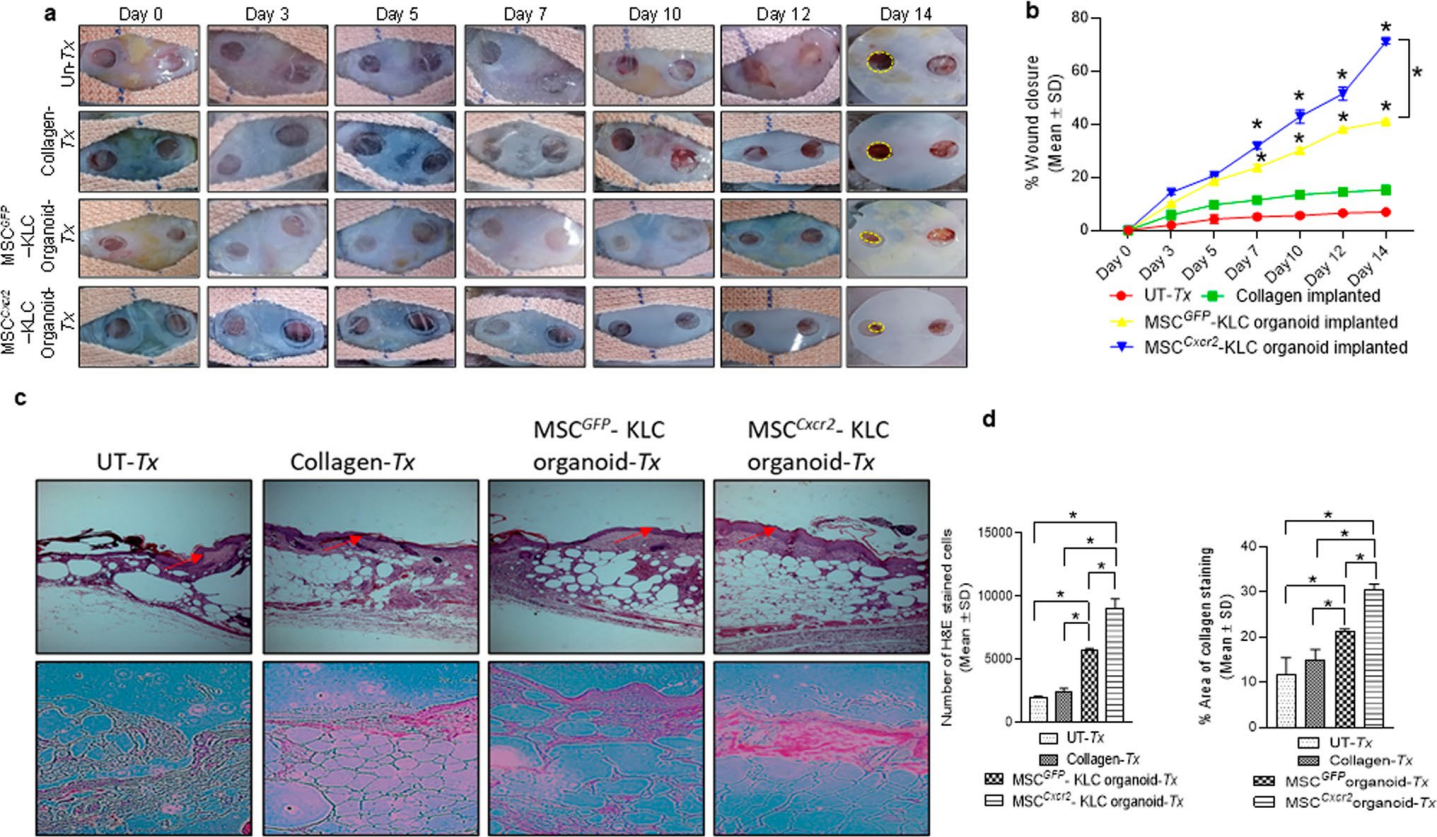
Accelerated wound closure with MSC^Cxcr2^-KLC organoid grafting in type 2 diabetic (db/db) transgenic mice **a** Morphometric analysis of representative images of *db/db* type 2 diabetic wounds from post-surgery day 0 to day 14. **b** Graph depicting a significant increase in wound closure in the MSC^*Cxcr2*^-KLC organoid grafted group from day 7 to day 14. **c** Representative images of hematoxylin and eosin (H&E) staining (upper panel) and Sirius red staining (lower panel) in regenerated *db/db* type 2 diabetic mice wound tissue (red arrow indicating the epidermal region of the regenerated skin tissue sections). **d** Quantitation of H&E-stained cells (left panel) indicating increased granulation in MSC^*Cxcr2*^-KLC organoid grafted group, and Sirius red staining (right panel) depicting a significant increase in percent area of collagen-stained tissue in MSC^*Cxcr2*^-KLC organoid grafted group. (*n* = 3, *p < 0.05 as compared with Un-*T*x/Collagen implanted/Tx group/MSC^*GFP*^-KLC organoid in morphometric analysis and histology quantification)

**Fig. 8:**
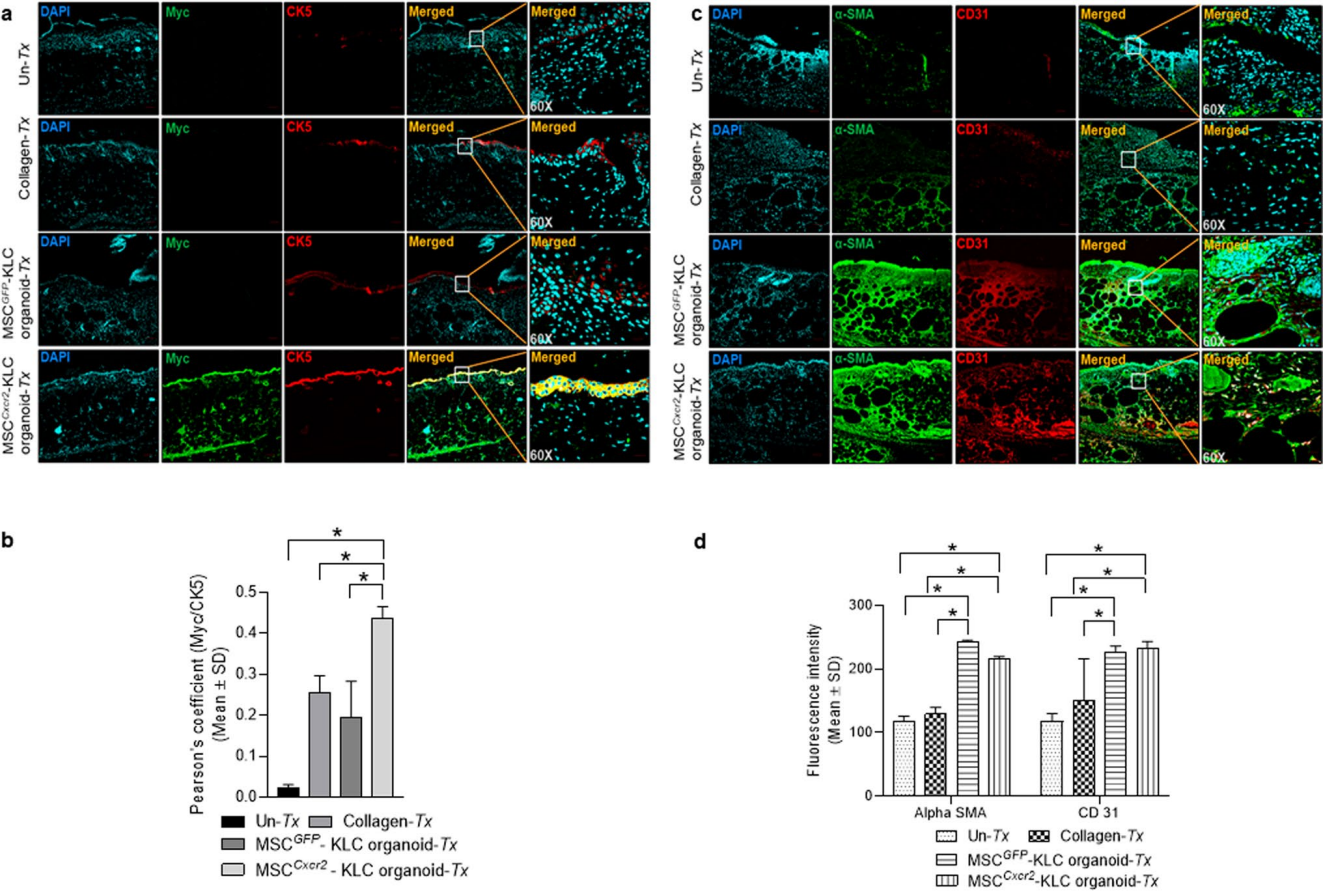
Enhanced re-epithelialization and vascularisation at type 2 diabetic mice wound bed **a** Representative confocal microscopy images and **b** quantification of Pearson’s correlation coefficient of *db/db* type 2 diabetic mice wound tissue immunostained with Myc tag and CK5 depicting significantly higher colocalization in MSC^*Cxcr2*^-KLC organoid grafted group. **c** Representative confocal microscopy images and **d** quantification of fluorescence intensity of *db/db* type 2 diabetic regenerated tissue sections immunostained with α-SMA and CD31 depicting significantly increased staining of these markers in both MSC^*GFP*^-KLC and MSC^*Cxcr2*^-KLC organoid grafted groups. (*n* = 3, *p < 0.05 as compared with UT/Collagen implanted/Tx/MSC^*GFP*^-KLC for re-epithelialization and Un-*Tx*/Collagen implantation for vascularization) (Scale bar: 10×–100 µm, 60×–20 µm)

The conventional 2D cell culture often fails to represent the structural and physiological properties of a complex structure like skin. Therefore, 3D culture systems are warranted for depicting the pathophysiology of various skin diseases and to generate skin equivalents [24, 25] that can be used for grafting/transplantation in chronic non-healing wounds. This led us to evaluate the efficiency of MSC^*Cxcr2*^.KLC to form stratified 3D skin organoids. The MSC^*Cxcr2*^.KLC or the MSC^*GFP*^.KLC (control) was seeded onto the fibroblast-derived dermal equivalent as described in the flow chart (Fig. 6a) and Materials and Methods. A marked increase in the expression of the keratinocyte markers—*Ck1*, *Ck13*, and *Sfn*, and the epithelial markers—*Cdh1* and *Muc1* was observed in the MSC^*Cxcr2*^-KLC organoids as compared with the MSC^*GFP*^-KLC organoids (Fig. 6b). Further, the organoid whole mounts were subjected to H&E staining to determine the morphology (Fig. 6c), and co-immuno-stained with Myc tag and CK5 (Fig. 6d, upper panel), and Involucrin and CK14 (Fig. 6d, lower panel) A significantly increased expression, represented by the increased fluorescence intensity of the markers, was observed in MSC^*Cxcr2*^-KLC organoids as compared to the MSC^*GFP*^-KLC organoids (Fig. 6d, e). Next, these skin organoids were grafted in the chronic excisional splinting wound model generated in type 2 diabetic (*db/db*) transgenic old mice which correlates well with the occurrence of DFU in patients with diabetes mellitus at an advanced age [26]. Michaels et al. demonstrated significant impairment in vascularity, cellular proliferation, and granulation tissue formation at the wound bed of *db/db* transgenic mice representing type 2 diabetes as compared with the STZ-induced mice model of type 1 diabetes leading to delayed wound healing in the former than later [27]. Interestingly, the percent wound closure observed in the present study in the un-transplanted group (Un-Tx) in *db/db* mice was less (6.9%) (Fig. 7a, b) as compared to the Un-Tx group in the type1 diabetic model (12.6%) (Fig. 4b, c) indicating the severity of type 2 diabetic wounds. Morphometric wound healing analysis depicted a marked decrease in wound size (Fig. 7a) and a significant increase in the percent wound closure in the MSC^*Cxcr2*^-KLC organoid grafted group as compared with the Un-Tx, empty collagen grafted, and MSC^*GFP*^-KLC organoid grafted groups (Fig. 7b). Although we observed a significant increase in the control MSC^*GFP*^-KLC organoid grafted group (41.1% wound healing) as compared with the other control groups (Un-*Tx* and empty collagen grafted groups), however, it was less effective as compared with the MSC^*Cxcr2*^-KLC organoid grafted group which showed $\sim$ 71% wound healing (Fig. 7a, b). These results were supported by histological analysis of the hematoxylin–eosin-stained skin tissue sections which depicted increased re-epithelialization and thickness of the epidermis along with Sirius red staining depicting increased collagen deposition in the MSC^*Cxcr2*^-KLC organoid grafted group (Fig. 7c, d). Additionally, immunofluorescence analysis revealed a significantly higher co-localization of CXCR2 with the Myc tag in the MSC^*Cxcr2*^-KLC organoid grafted group as compared with the MSC^*GFP*^-KLC organoid grafted group (Fig. S10a, b). Similarly, immunostaining with GFP and CXCR2 revealed a higher immunofluorescence intensity of GFP in the MSC^*GFP*^-KLC organoid grafted group and CXCR2 in the MSC^*Cxcr2*^-KLC organoid grafted group (Fig. S10c,d). Immunofluorescence analysis revealed a marked increase in the expression of pERK1/2 (Fig. S11a) and pSTAT3 (Fig. S11b) in the MSC^*Cxcr2*^-KLC,derived 3D skin organoid grafted group. Further, increased co-localization of Myc/pERK1/2 (Fig. S11c) and Myc/pSTAT3 (Fig. S11d) was also observed in the regenerated tissue sections of MSC^*Cxcr2*^-KLC organoid grafted group suggesting the activation of ERK1/2 and STAT3 signaling pathways. Next, co-immunostaining of the Myc tag with the keratinocyte markers CK5 (Fig. 8a, b) and Involucrin (Fig. S12a, b) also depicted higher co-localization in the MSC^*Cxcr2*^-KLC organoid grafted group further confirming accelerated chronic type 2 diabetic wound tissue regeneration by re-epithelialization. Similarly, immunostaining with GFP and CK5 (Fig. S13a, b) and GFP and IVL (Fig. S13c, d) revealed a higher immunofluorescence intensity of GFP in the MSC^*GFP*^-KLC organoid grafted group and CK5 and IVL in the MSCCxcr2-KLC organoid grafted group (Fig. S13c, d). Further, co-immunostaining of α-SMA and CD31 (Fig. 8c, d) depicted a higher expression in the dermal layer of both the MSC^*Cxcr2*^-KLC organoid and MSC^*GFP*^-KLC organoid grafted groups indicating enhanced vascularity or endothelialization-mediated skin tissue regeneration in type 2 diabetic wound bed. Fibroblasts secrete several angiogenic growth factors, such as VEGF, TGF-β, and PDGF, which support the formation of endothelial cell lumen and endothelial cell sprouting [28]. Since we plated an equal number of fibroblasts in the dermal equivalent of the organoid, we achieved an equal extent of vascularization in both the groups but enhanced re-epithelialization as well as re-endothelialization-mediated skin tissue regeneration in the MSC^*Cxcr2*^-KLC organoid grafted group.

## Discussion

The complex process of wound healing is severely disrupted due to the pathological abnormalities associated with chronic diseases like diabetes. Diabetic foot ulcers demonstrate altered expression of chemokines, cytokines, and growth factors that regulate the process of wound healing [6–8]. Among the stem cell therapies, MSC transplantation in diabetic wound healing has shown promising results. However, low engraftment, survivability, proliferation, and differentiation of the transplanted MSCs are challenging problems that limit their clinical translation [13–15]. This necessitates developing bioengineered MSCs that enhance their engraftment, and trans-differentiation upon transplantation in the chronic wound microenvironment. MSCs express a wide repertoire of chemokine receptors that modulate their physiological responses in the presence of their cognate ligands [12]. The chemokine ligand/receptor axis-based gene therapy approach has been widely used to accelerate wound closure. Our previous study reported an enhanced migration of the intravenously transplanted *Cxcr6* bioengineered MSCs in response to the increased level of its cognate ligand CXCL16 at the wound area [16]. The overexpression of *Cxcr4*, a potent angiogenic factor, also enhanced the migration of MSCs and angiogenesis at the injury site via its cognate ligand CXCL12 [29]. Similarly, CCR2 overexpressed MSCs exhibited increased homing toward the injury site in response to the increased level of CCL2 in diabetic db/db mice wounds [10]. In this line, we have observed a significant increase in the expression of the CXCR2 cognate ligand CXCL2 in type 1 and type 2 diabetic wounds as compared with control non-wounded skin (Fig. S14). CXCR2 has been known to promote the recruitment of neutrophils at the site of inflammation and facilitate wound healing and angiogenesis. In the present study, we demonstrated the role of the chemokine receptor CXCR2 in regulating MSC physiology. Recently, *Cxcr2* overexpression in MSCs has also been demonstrated to improve the migration and homing of the cells toward the site of injury in response to the ligands CXCL2 and CXCL5 [30, 31]. However, we did not observe any significant change in MSC migration with *Cxcr2* overexpression in the absence or presence of the ligand CXCL2. Interestingly, the observed increase in proliferation of MSCs with *Cxcr2* overexpression in our study correlates well with the recent study by Shen et al. which demonstrated an enhanced survival rate of the *Cxcr2* overexpressed MSCs both in vitro and in vivo [31]. Our study further revealed the role of pSTAT3 and pERK1/2 in regulating CXCR2.mediated transcriptional regulation of the downstream target, *FGFR2IIIb*. ERK1/2 has been known to be a major downstream effector in CXCR2-mediated signal transduction [31, 32]. *FGFR2IIIb*, also known as *FGFR2b* or *KGFR* has been reported to regulate the early differentiation of normal human keratinocytes [33].

Re-epithelialization is a crucial process regulated by keratinocytes in which the dermal and mucosal wound surface is covered, and the epidermal barrier is restored. Several studies have reported impaired keratinocyte proliferation and migration in diabetic patients [34, 35] as well as in diabetic animal models [36, 37]. In the present study, we demonstrated the role of CXCR2 in enhancing the transdifferentiation of MSCs toward keratinocyte-like cells. Subsequently, KGF-induced enhanced proliferation and migration of MSC^*Cxcr2*^-KLCs correlated well with prior studies demonstrating KGF-mediated enhanced proliferation and migration of keratinocytes [38]. Interestingly, Guo et al. reported no significant change in the rate of wound healing in KGF knockout mice as compared to control [39]. However, a more recent study by the same group reported a significantly delayed wound closure rate in diabetic mice in the absence of KGF (FGF7) as compared to the non-diabetic group, highlighting the importance of KGF/FGFR2IIIb signaling in accelerating wound closure and re-epithelialization in diabetic mice [40]. Furthermore, delayed or impaired wound healing was reported in CXCR2 deficient mice due to the lack of neutrophil and monocyte recruitment and reduced secretion of IL-1β at the wound site [41, 42]. Corroborating with these observations, our study demonstrated enhanced re-epithelialization-mediated skin tissue regeneration in murine diabetic wounds upon transplantation of *Cxcr2*-bioengineered MSCs. The higher engraftment of MSC^*Cxcr2*^ at the diabetic wound bed in our preclinical study as well as others was in part due to an increased expression of its cognate ligand CXCL2 in diabetic conditions [43]. Our observations correlated well with a study by Kroeze et al., which identified CXCR2 along with four other chemokine receptors CCR1, CCR10, CXCR1, and CXCR3 to be involved in the autocrine regulation of re-epithelialization [44].

Skin is a structurally complex organ performing multiple functions and mimicking the morphological and physiological properties of skin that require 3D culture systems which resemble the in vivo systems. This led us to generate a 3D organotypic spheroid culture for the transdifferentiation of these bioengineered MSCs. Recent studies have focused on generating functional epidermal spheroids that demonstrate vasculogenic properties and the growth of skin appendages like hair follicles [20, 45]. Furthermore, we utilized air–liquid interface culture in our study settings to generate a 3D stratified skin organoid as reported earlier by layered co-culture of fibroblasts and keratinocytes that successfully generates 3D stratified skin structures [21, 45, 46]. We generated the dermal equivalent by seeding NIH3T3 cells in a collagen scaffold and subsequently seeded the bioengineered MSC,derived KLCs on the dermal equivalent. Diabetic foot ulcers are not only more prevalent among older patients with type 2 diabetes but also the vascularity is poor in the lower extremities of type 2 diabetic patients which leads to non-healing wounds [47]. Grafting of the *Cxcr2*-bioengineered MSC,derived KLC skin organoids at the chronic wounds in old (50–52 weeks old) transgenic *db/db* mice in the present study resulted in an enhanced re-epithelialization at the epidermal layer by the transdifferentiated KLCs as well as vascularity evidenced by increased staining of CD31 at the dermal layer.

In conclusion, we demonstrated the role of the chemokine receptor *Cxcr2* in enhancing the transdifferentiation potential of MSCs toward keratinocyte-like cells via transcriptional upregulation of *FGFR2IIIb*. These bioengineered MSC^*Cxcr2*^. KLC, derived skin organoids upon grafting onto chronic diabetic wounds potentiated the healing by skin regeneration. Our study demonstrates a platform technology for chronic diabetic wound healing (diabetic foot ulcers) with allogenic MSC,derived skin organoid grafts.

### Supplementary Information

Below is the link to the electronic supplementary material.

Supplementary file1 (DOCX 6115 kb)

Supplementary file2 (TIF 72 kb)

Supplementary file3 (TIF 603 kb)

Supplementary file4 (TIF 1049 kb)

Supplementary file5 (TIF 589 kb)

Supplementary file6 (TIF 492 kb)

Supplementary file7 (TIF 24 kb)

Supplementary file8 (TIF 515 kb)

Supplementary file9 (TIF 162 kb)

Supplementary file10 (TIF 364 kb)

Supplementary file11 (TIF 378 kb)

Supplementary file12 (TIF 96 kb)

Supplementary file13 (TIF 108 kb)

Supplementary file14 (TIF 941 kb)

Supplementary file15 (TIF 788 kb)

## Data Availability

All the data generated have been included in the manuscript and supplemental data available online.
