# Supplementary material for: Bioengineered MSCCxcr2 transdifferentiated keratinocyte-like cell-derived organoid potentiates skin regeneration through ERK1/2 and STAT3 signaling in diabetic wound
Source: Cell Mol Life Sci. 2024 Apr 10;81(1):172. doi: 10.1007/s00018-023-05057-3 (PMC11006766; doi:10.1007/s00018-023-05057-3)
Supplement: Supplementary file 1 — Supplementary file1 (DOCX 6115 kb) [file 18_2023_5057_MOESM1_ESM.docx]

**Supplemental material**

**Supplemental table**

**Table ST1. List of expression primers of mouse-gene specific markers.**

| S.No. | Name (Species: Mouse) | Primer | Sequence |
| --- | --- | --- | --- |
| 1 | *Cxcr2* | FP | 5’AGCAGAGGATGGCCTAG 3’ |
|  |  | RP | 5’TCCACCTACTCCCATTTG 3’ |
| 2 | *Bnc1* | FP | 5’CTATCCCTGTTCGCCTGAAA 3’ |
|  |  | RP | 5’ACTGCTGCAAGGTAGCCACT 3’ |
| 3 | *Ck5* | FP | 5’AGCTGAGGAACATGCAGGAC 3’ |
|  |  | RP | 5’GGCAGCATCCACATCCTTCT 3’ |
| 4 | *Ck14* | FP | 5’AGAACCTCAATGACCGCCTG 3’ |
|  |  | RP | 5’GCTGCCAGGATCTTGCTCTT 3’ |
| 5 | *Ck13* | FP | 5’GAGAAGATCACCATGCAGAACC 3’ |
|  |  | RP | 5’GTGGCTTCCAGAATCTTGATCCG 3’ |
| 6 | *Ck1* | FP | 5’TGCAAGAAGCAGATCTCCCAA 3’ |
|  |  | RP | 5’TGGAAGTCACGCAGCAATCT 3’ |
| 7 | *Ck10* | FP | 5’CAGCTGGCCCTGAAACAATC 3’ |
|  |  | RP | 5’AGTTGTTGGTACTCGGCGTT 3’ |
| 8 | *Flg* | FP | 5’TCAGCTGACAGGCAAGGG 3’ |
|  |  | RP | 5’GACTCCTCCTCGCTGTGT 3 |
| 9 | *Sfn* | FP | 5’CGTCCCAGCCCTTTTACCTT 3’ |
|  |  | RP | 5’AGAGTAACGCTGGGTTCTGC 3’ |
| 10 | *Ivl* | FP | 5’TGAACCAGAACTGCAACTGG 3’ |
|  |  | RP | 5’ CTTTCTGATCCCCTGCCATA 3’ |
| 11 | *EpCam* | FP | 5’GCTGTCATTGTGGTGGTGTC 3’ |
|  |  | RP | 5’ATCTGCAGTCCGAGCTCTTC 3’ |
| 12 | *Cdh1* | FP | 5’CGGAGAGGAGAGTCGAAGTG 3’ |
|  |  | RP | 5’CATGCTCAGCGTCTTCTCTG 3’ |
| 13 | *Cldn1* | FP | 5’AGGTCTGGCGACATTAGTGG 3’ |
|  |  | RP | 5’CGTGGTGTTGGGTAAGAGGT 3’ |
| 14 | *Muc1* | FP | 5’GACATCTTTCCAACCCAGGA 3’ |
|  |  | RP | 5’GGGGTGACTTGCTCCTACAA 3’ |
| 15 | *Nestin* | FP | 5’AAGACCAGCAGGCGTTTAGA 3’ |
|  |  | RP | 5’TCCTCTGCGTCTTCAAACCT 3’ |
| 16 | *PodXl* | FP | 5’CGAGAGGCAGACAGGATTTC 3’ |
|  |  | RP | 5’TGCACAAGAAGGAACACTGG 3’ |
| 17 | *CD73* | FP | 5’GATCCGCAAGGAAGAACCCA 3’ |
|  |  | RP | 5’ATGGCCATAGCATCGTAGCC 3’ |
| 18 | *CD90.2* | FP | 5’CCAATGAGGATGAGGGCTTA 3’ |
|  |  | RP | 5’GCAGGCTCGTGTTTTAGAGG 3’ |
| 19 | *CD105* | FP | 5’CTTCCAAGGACAGCCAAGAG 3’ |
|  |  | RP | 5’GTGGTTGCCATTCAAGTGTG 3’ |
| 20 | *CD49f* | FP | 5’GCCTTTTCACTGGACTCAGG 3’ |
|  |  | RP | 5’CCACCACTGCCACATCATAG 3’ |
| 21 | *Cxcl2* | FP | 5’TCCAGAGCTTGAGTGTGACG 3’ |
|  |  | RP | 5’CTTTGGTTCTTCCGTTGAGG 3’ |
| 22 | *Eu 18S rRNA* | FP | 5’AAACGGCTACCACATCCAAG 3’ |
|  |  | RP | 5’CCTCCAATGGATCCTCGTTA 3’ |

(FP – forward primer, and RP – reverse primer).

**Table ST2. List of primary and secondary antibodies used in immunofluorescence immunohistochemical analysis.**

| **S. No.** | **Name** | **Manufacturer (Cat. No.)** |
| --- | --- | --- |
|  | Anti-Involucrin antibody | Merck (SAB4200794) |
|  | Anti-Keratin 14 antibody | Merck (SAB4501657) |
|  | Anti-Keratin 5 antibody | Merck (SAB4501651) |
|  | Anti Myc-Tag | CST (2276S) |
|  | Anti-CXCR2 antibody | Invitrogen (PA5102942) |
|  | Anti-α-SMA antibody | Sigma  (A2547) |
|  | Anti-CD31 antibody | CST (77699S) |
|  | Anti-p-Tyr antibody | Biolegend (309302) |
|  | Anti-FGFR2IIIb antibody | R&D (133730) |
|  | Anti-GFP antibody | CST (2955S) |
|  | Anti-GFP antibody | Puregene (PG-41002) |
|  | Anti-pERK antibody | CST (4370S) |
|  | Anti-ERK antibody | CST (4695S) |
|  | Anti-pSTAT3 antibody | CST (9145S) |
|  | Anti-STAT3 antibody | CST (12640S) |
|  | Anti-mouse Alexa Fluor 488 | Invitrogen (A-21121) |
|  | Anti-rabbit Alexa Fluor 488 | Invitrogen (A-11070) |
|  | Anti-mouse Alexa Fluor 555 | Invitrogen (A-21422) |
|  | Anti-rabbit Alexa Fluor 555 | Invitrogen (A-31572) |

**Supplemental figures**

***
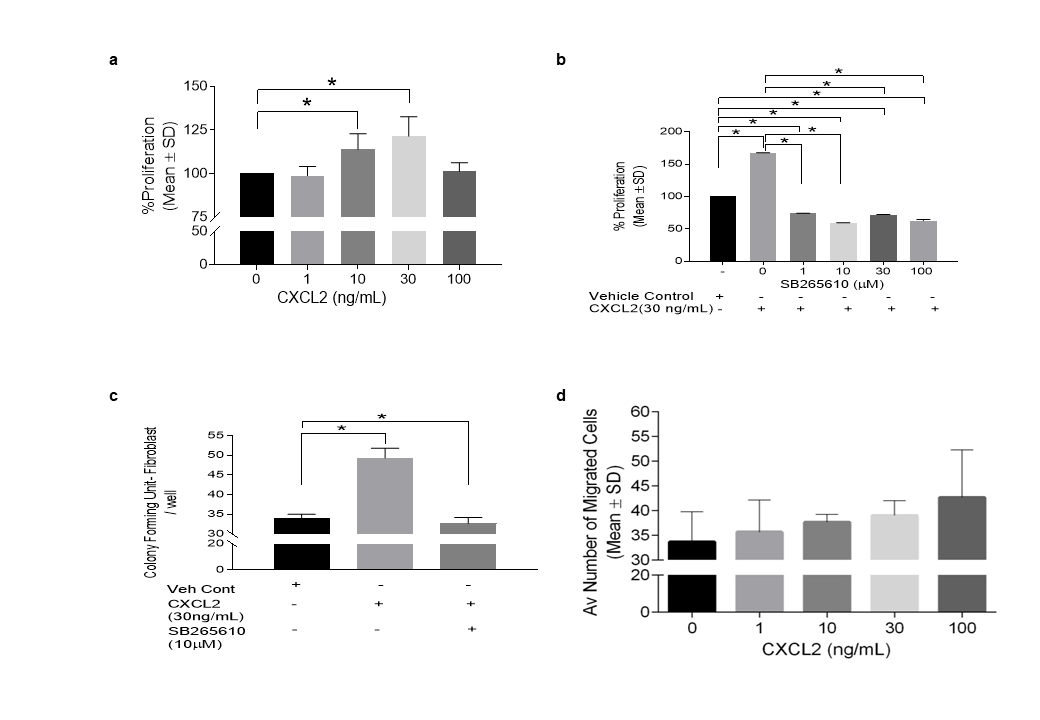
***

***Figure S1. CXCL2 induces the proliferation of MSCs.*** (a) A concentration-dependent increase in MSC proliferation was observed in the presence of CXCL2. (b) CXCR2 pharmacological inhibitor, SB265610 depicted a significant decrease in CXCL2 mediated MSC proliferation. (c) CXCL2-induced colony-forming efficiency of MSCs was abrogated in the presence of a CXCR2 blocker, SB265610. (d) CXCL2 did not affect the migratory capacity of MSCs as evidenced by the insignificant difference in the number of cells migrated towards the lower chamber of the Boyden chamber migration assay system. (n=3, *p<0.05 as compared with Veh. Cont. / CXCL2 treated group).


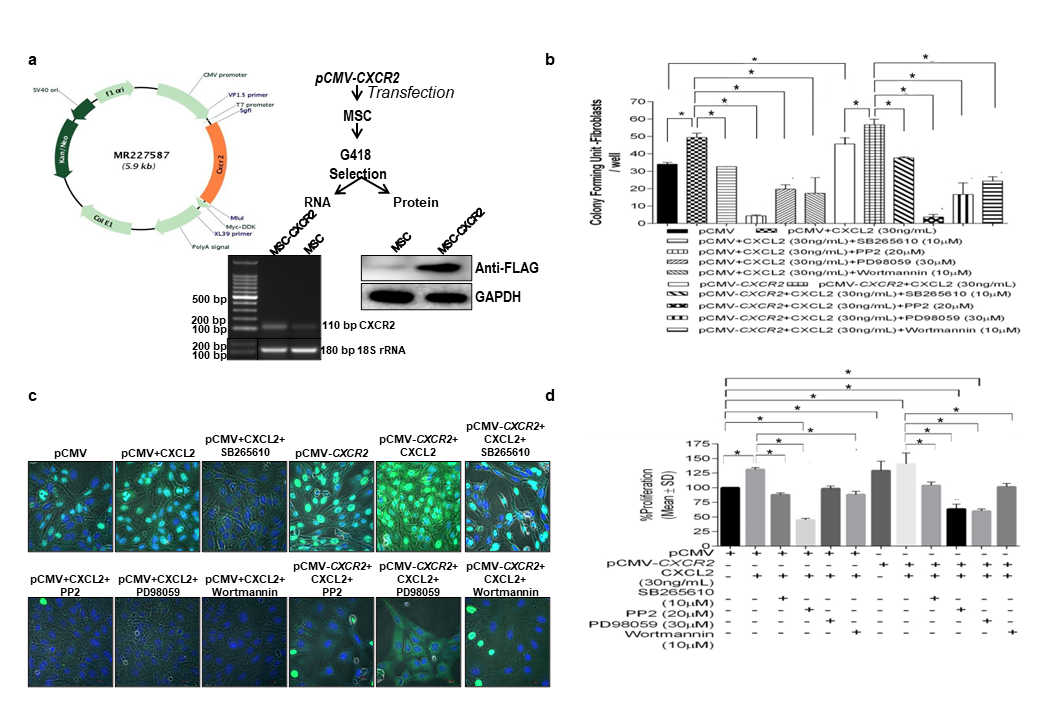
***Figure S2. Cxcr2 overexpression in MSCs induces proliferation.*** (a) Schematic representation of the approach for transient overexpression of *Cxcr2* in MSCs including transfection of MSCs, followed by G418 selection, and confirmation of overexpression using RT-PCR and immunoblot analysis. (b) *Cxcr2* overexpression significantly induced the colony-forming efficiency which was further potentiated with CXCL2. CXCR2 pharmacological blocker SB265610, along with the signaling molecule inhibitors, PP2 (Src), PD98059 (Erk1/2), and Wortmannin (Akt) significantly reverted the CXCL2-induced colony formation. (c) Representative confocal images of BrdU incorporation in MSCs transfected with *pCMV-eGFP* (control) or *pCMV-Cxcr2-MycDDK*. CXCL2 treatment increases BrdU incorporation in both control and *Cxcr2* overexpressed cells which were abrogated by SB265610, Wortmannin, PP2, and PD98059. (d) BrdU incorporation was also quantitated and based on the absorbance and calculated as percent proliferation. CXCL2-induced proliferation was inhibited in both control and *Cxcr2*-overexpressed MSCs in the presence of signaling inhibitors. (n=3, *p<0.05 as compared with Veh. Cont. / CXCL2 treated group).

**
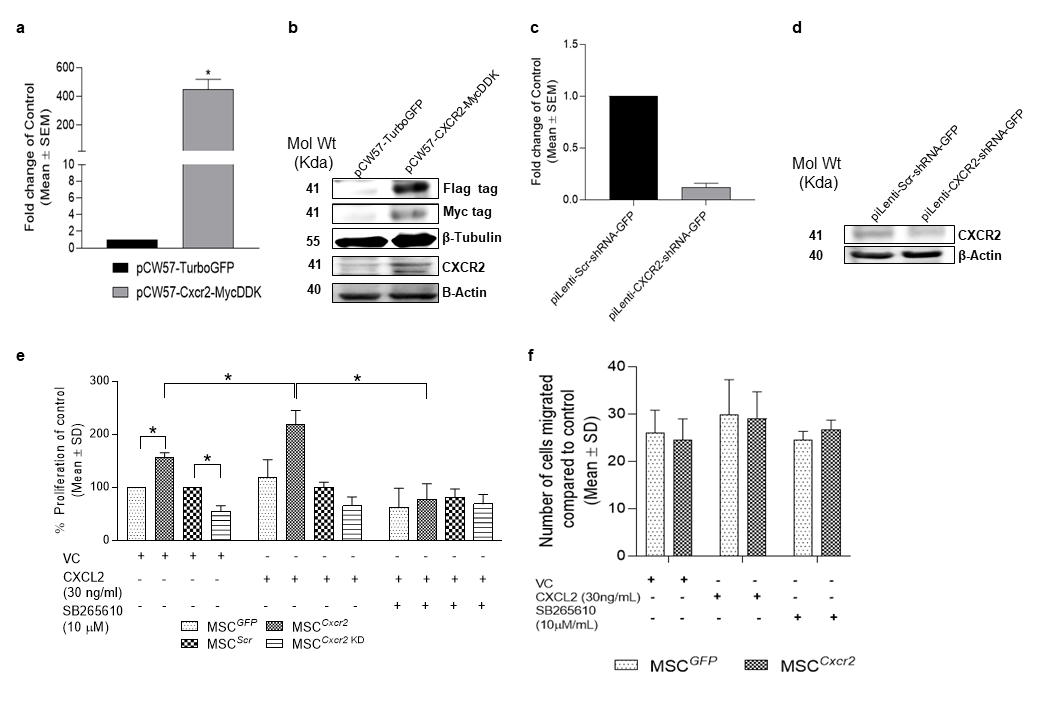
**

***Figure S3. Generation of stable MSCs with Cxcr2 overexpression and/or silencing.*** (a) qRT-PCR and (b) immunoblot analysis confirming the stable overexpression of *Cxcr2-Myc* in MSCs. Similarly, (c) qRT-PCR and (d) immunoblot analysis confirmed *Cxcr2* silencing in MSCs. (e) Graph depicting *Cxcr2*-mediated increased proliferation in MSCs which was further increased in the presence of CXCL2 and abrogated in the presence of CXCR2 inhibitor SB265610. (f) Graph depicting no change in the migratory potential of MSCs with *Cxcr2* modulation. (n=3, *p<0.05 as compared with MSC*^GFP^* / MSC*^Scr^* / MSC*^Cxcr2^* / MSC*^Cxcr2^* + CXCL2).

***
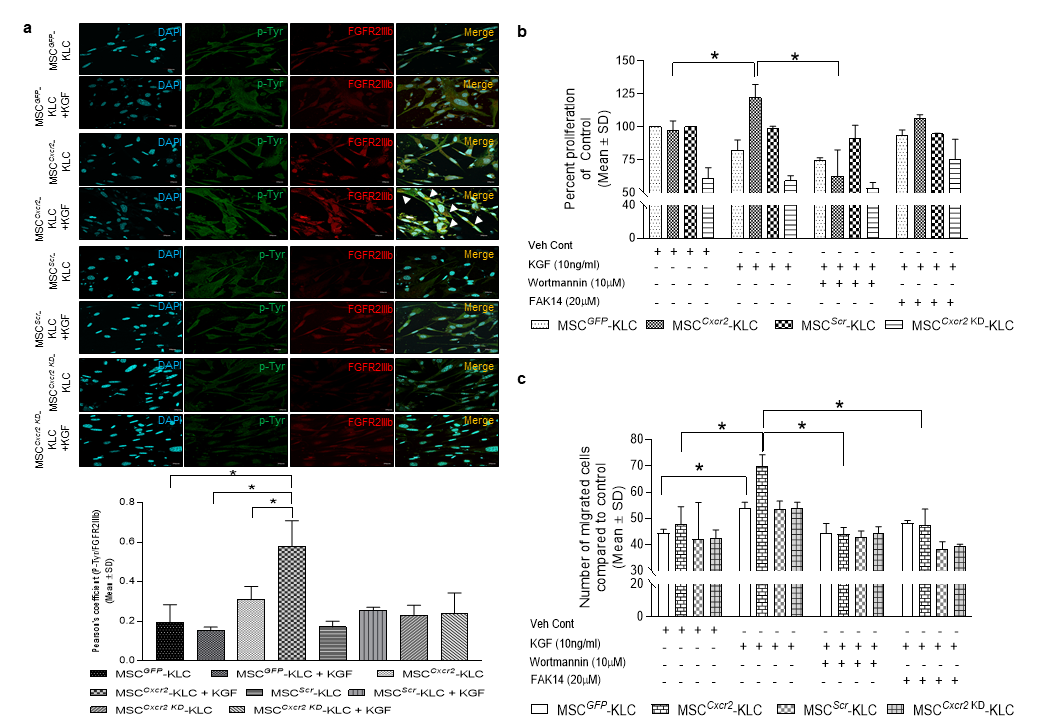
***

***Figure S4. KGF potentiates the keratinocyte-physiology of MSC^Cxcr2^-derived KLCs****.* (a) *The upper panel* depicts the confocal microscopy images of MSC*^GFP^*, MSC*^Cxcr2^*, MSC*^Scr^*, and MSC*^Cxcr2 KD^* -derived KLCs in the absence or presence of KGF (10 ng/ml). *Lower panel* MSC*^Cxcr2^*-KLCs showed a significant increase in Pearson’s correlation coefficient in the presence of KGF suggesting a significant increase in the colocalization of P-Tyr and FGFR2IIIb thereby suggesting FGF7/KGF-induced activation of FGFR2IIIb. (n=3, p < 0.05 as compared with *MSC*^GFP^*-KLC / MSC*^GFP^*-KLC + KGF / MSC*^Cxcr2^*-KLC). Graphs depicting significantly increased (b) proliferation and (c) migration of MSC*^Cxcr2^*-KLCs in the presence of KGF (10ng/ml) that was differentially reverted significantly in the presence of Wortmannin (10 µM), and FAK14 (20 µM). (n=3, *p < 0.05 as compared with MSC*^Cxcr2^*-KLC / MSC*^Cxcr2^*-KLC + KGF).

***
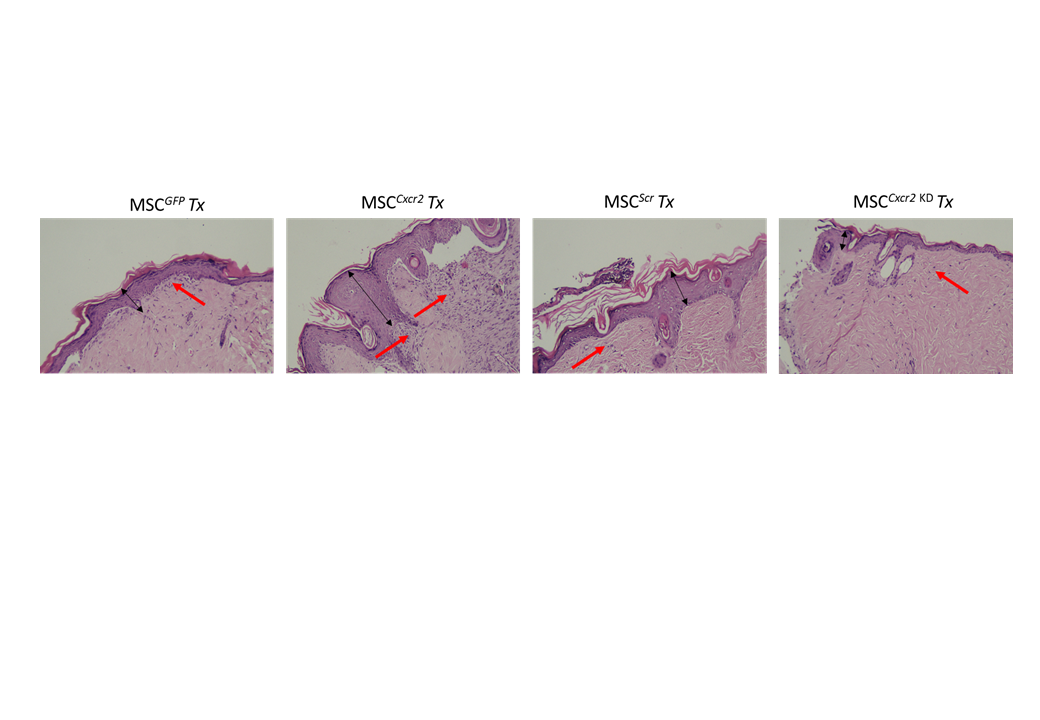
***

***Figure S5. H&E staining of the regenerated tissue sections.*** Representative images of H&E-stained tissue sections depicting a marked increase in the infiltration of H&E-stained cells (indicated by red arrow), and epidermal thickness (indicated by black arrow) at the regenerated wound site in MSC*^Cxcr2^* transplanted group.

***
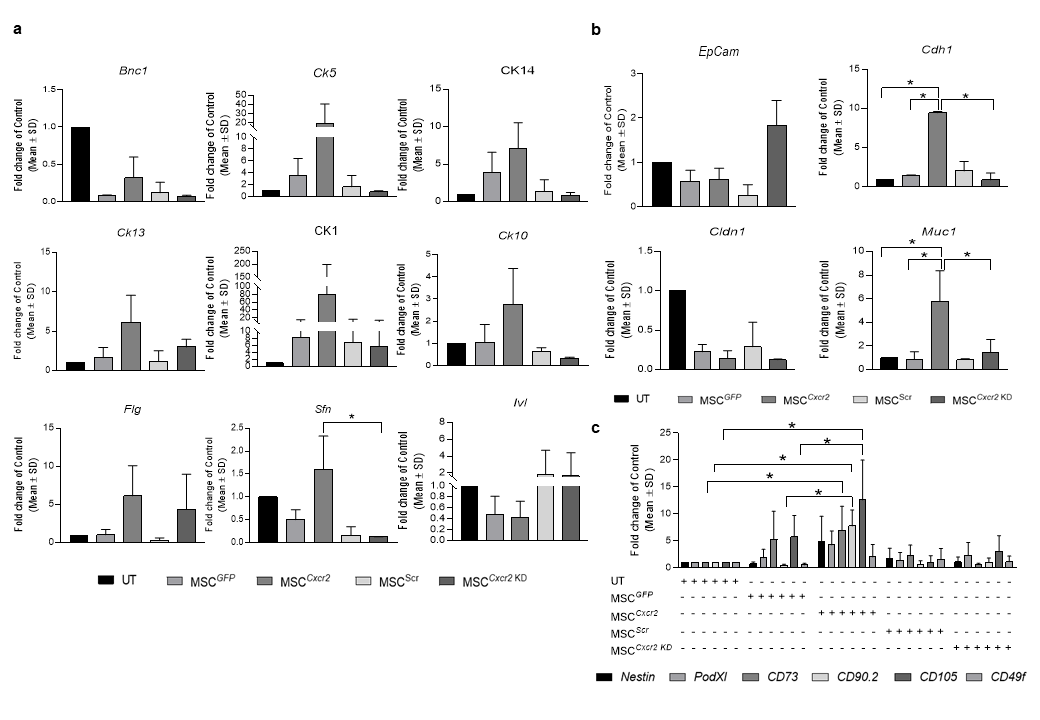
***

***Figure S6. Expression of keratinocyte-, epithelial-, and mesenchymal-specific genes at the wound site on PS-day 7.*** Differential expression analysis of the (a) keratinocyte- and (b) epithelial-specific genes showing significant increase in the expression of E-Cadherin (*Cdh1*) and Mucin-1 (*Muc1*) in the regenerated type 1 diabetic wound tissues at PS-day 7. (c) Differential expression analysis of the mesenchymal genes at PS-day 7. (n=3, *p<0.05 as compared with UT / MSC*^GFP^* / MSC*^Cxcr2^*).


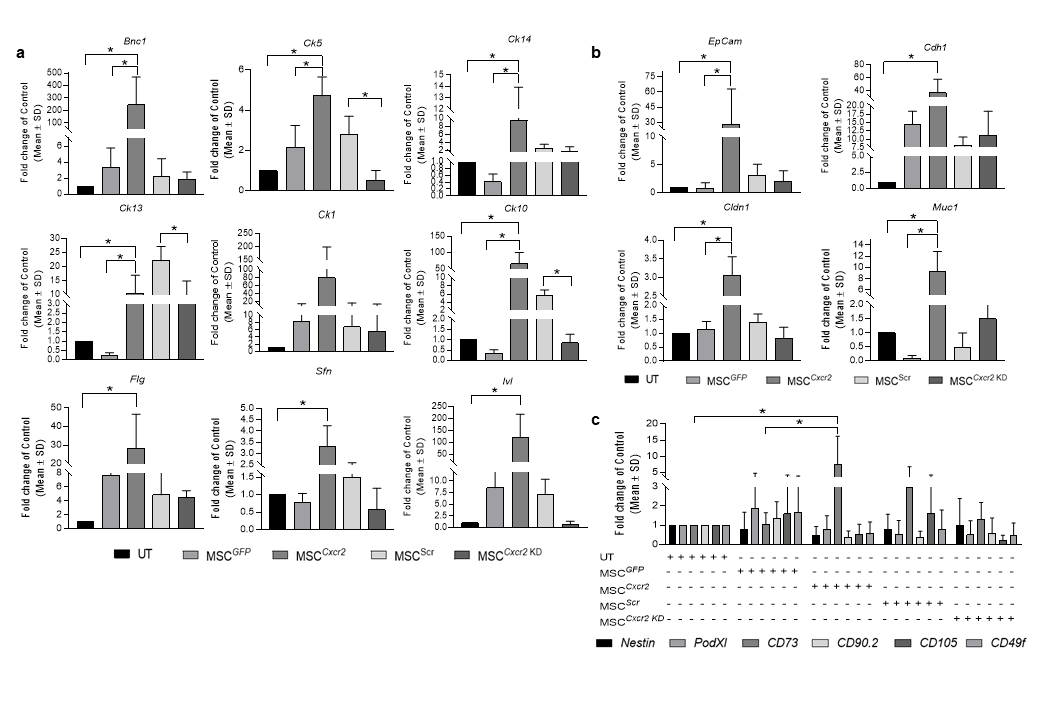
***Figure S7. Cxcr2-mediated enhanced re-epithelialization at type 1 diabetic mice wound bed.*** Differential expression analysis of the (a) keratinocyte- and (b) epithelial-specific genes in the regenerated type 1 diabetic wound tissues at PS-day 14 depicting significantly increased expression of the keratinocyte markers Basonuclin (*Bnc1*), Cytokeratin 5 (*CK5*), *CK14*, *CK13*, *CK10*, Filaggrin (*Flg*), Stratifin (*Sfn*), and Involucrin (*Ivl*) and epithelial markers *Ep-CAM*, E-Cadherin (*Cdh1*), Claudin (*Cldn1*), Mucin-1 (*Muc1*) in MSC*^Cxcr2^* transplanted group. (c) Differential expression analysis of the mesenchymal markers depicting reduced expression of *Nestin*, *Podxl*, *CD90.2*, *CD105*, and *CD49f* in MSC*^Cxcr2^* transplanted group. (n=3, *p<0.05 as compared with UT / MSC*^GFP^*).

***
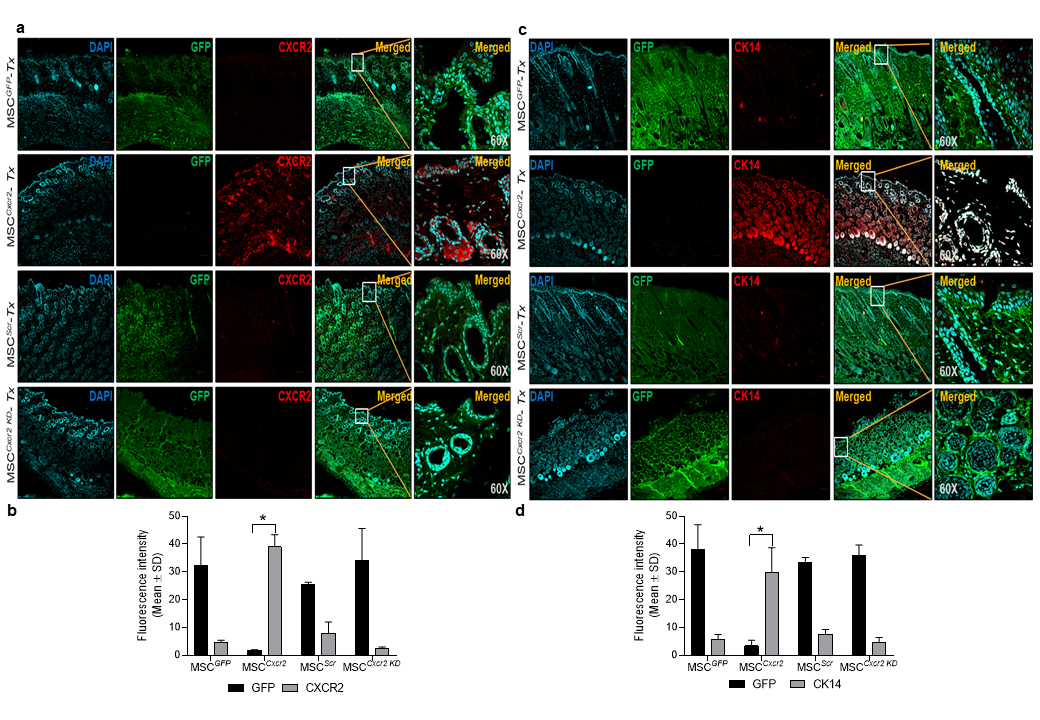
***

***Figure S8. Differential engraftment of GFP expressing cells at type 1 diabetic mice wound bed.*** (a) Representative confocal microscopy images of regenerated tissue sections immunostained with GFP and CXCR2 in MSC*^GFP^*, MSC*^Cxcr2^*, MSC*^Scr^*, and MSC*^Cxcr2^* ^KD^ transplanted groups. (b) Quantification of fluorescence intensity of GFP showed comparable engraftment of bioengineered MSCs at the wound site of these groups with significantly higher fluorescence intensity of CXCR2 in the MSC*^Cxcr2^* transplanted group as compared with others. (c) Similarly, representative confocal images of regenerated wound sections immunostained with GFP and CK14 in MSC*^GFP^*, MSC*^Scr^*, MSC*^Cxcr2^*, and MSC*^Cxcr2^* ^KD^ transplanted groups and (d) quantification of fluorescence intensity revealed comparable GFP but significantly higher CK14 in the MSC*^Cxcr2^* transplanted group. (n=3, *p<0.05 as compared with MSC*^GFP^*). (Scale bar: 10× - 100µm, 60× - 20µm).


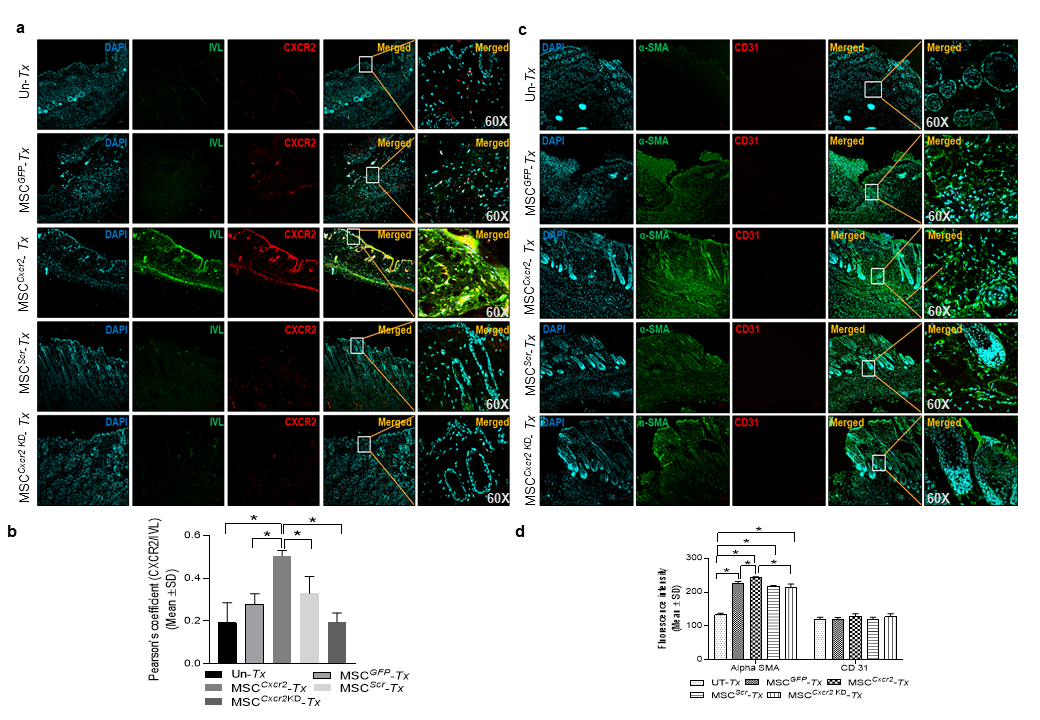
***Figure S9. Enhanced re-epithelialization but less vascularization at type 1 diabetic mice wound bed.*** Representative confocal microscopy images of regenerated tissue sections immunostained with (a) Involucrin and CXCR2 depicting significantly higher (b) colocalization in the MSC*^Cxcr2^* transplanted group. (n=3 p<0.05 as compared with *Un-*Tx* / MSC*^GFP^*-*Tx* / MSC*^Scr^*-*Tx* / MSC*^Cxcr2^* ^KD^-*Tx*). Similarly, representative confocal microscopy images of regenerated tissue sections immunostained with (c) α-SMA and CD31 depicted increased expression as evidenced by (d) higher fluorescence intensity of α-SMA but less expression of CD31 in all the transplanted groups. (n=3, *p<0.05 as compared with Un-*Tx* / MSC*^GFP^*-*Tx* / MSC*^Cxcr2^*-*Tx*). (Scale bar: 10× - 100µm, 60× - 20µm).

***
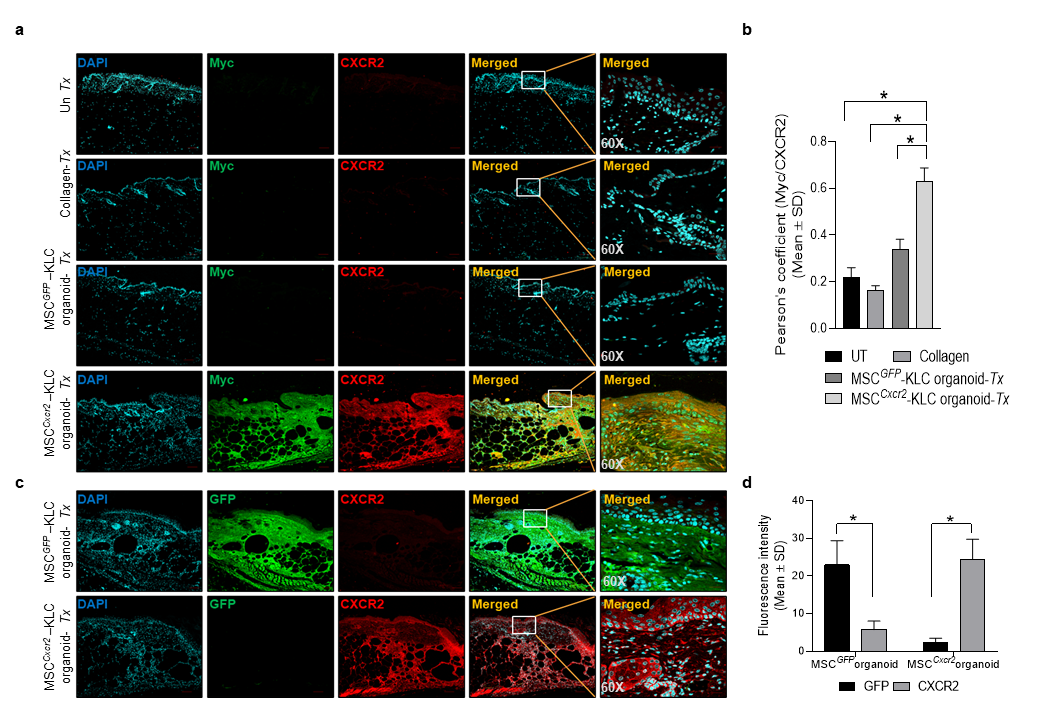
Figure S10. Colocalization of Myc-tag or GFP with Cxcr2 at type 2 diabetic mice wound bed grafted with bioengineered MSC-derived KLC organoids.*** Representative confocal microscopy images of regenerated tissue sections immunostained with (a) Myc tag and CXCR2 depicting significantly higher (b) colocalization in MSC*^Cxcr2^*-KLC organoid transplanted group while (c) GFP and CXCR2 coimmunostaining of the regenerated skin revealed comparable fluorescence intensities in the MSC*^GFP^*-KLC organoid and MSC*^Cxcr2^*-KLC organoid transplanted groups. (d) A higher fluorescence intensity of GFP in the former and CXCR2 in the later groups was observed. (n=3, *p<0.05 as compared with Un-*T*x / MSC*^GFP^*-KLC organoid / Collagen implanted groups). (Scale bar: 10× - 100µm, 60× - 20µm).

***
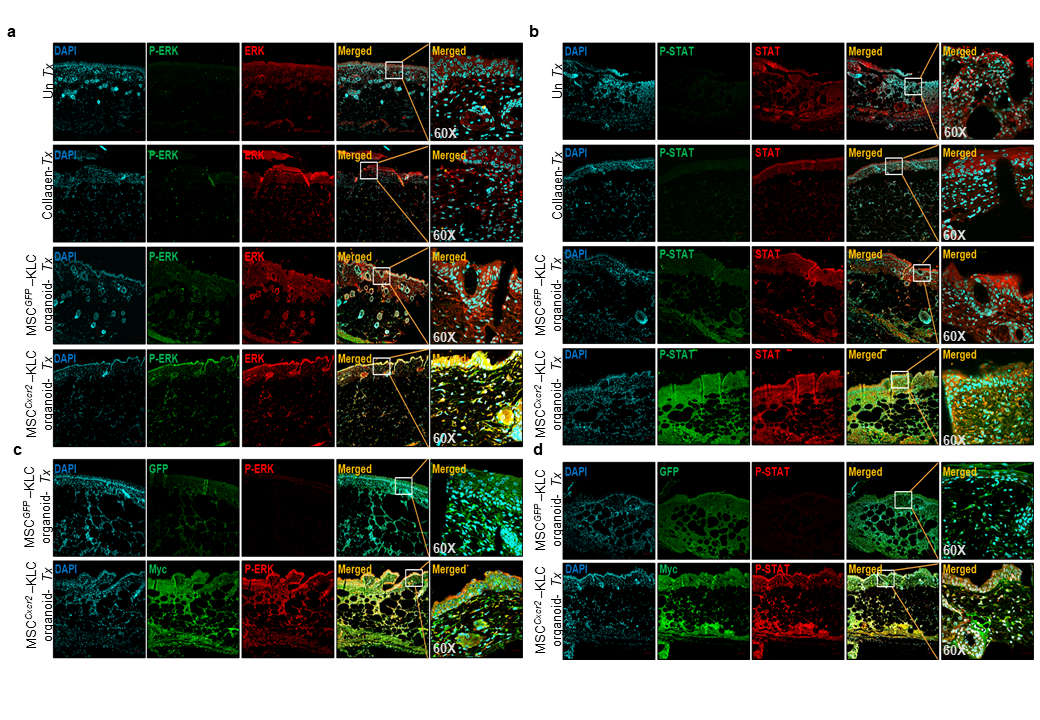
Figure S11.* *In vivo activation of ERK1/2 and STAT3 signaling pathways in MSC^Cxcr2^-KLC organoid grafted mice.*** Representative confocal microscopy images showing enhanced activation of (a) ERK1/2 (p-ERK1/2) and (b) STAT3 (p-STAT3) in the regenerated tissue sections of MSC*^Cxcr2^*-KLC organoid grafted mice. (c) Enhanced colocalization of Myc and p-ERK1/2 in MSC*^Cxcr2^*-KLC organoid grafted group. (d) Enhanced colocalization of Myc and p-STAT3 in MSC*^Cxcr2^*-KLC organoid grafted group. (Scale bar: 10× - 100µm, 60× - 20µm).


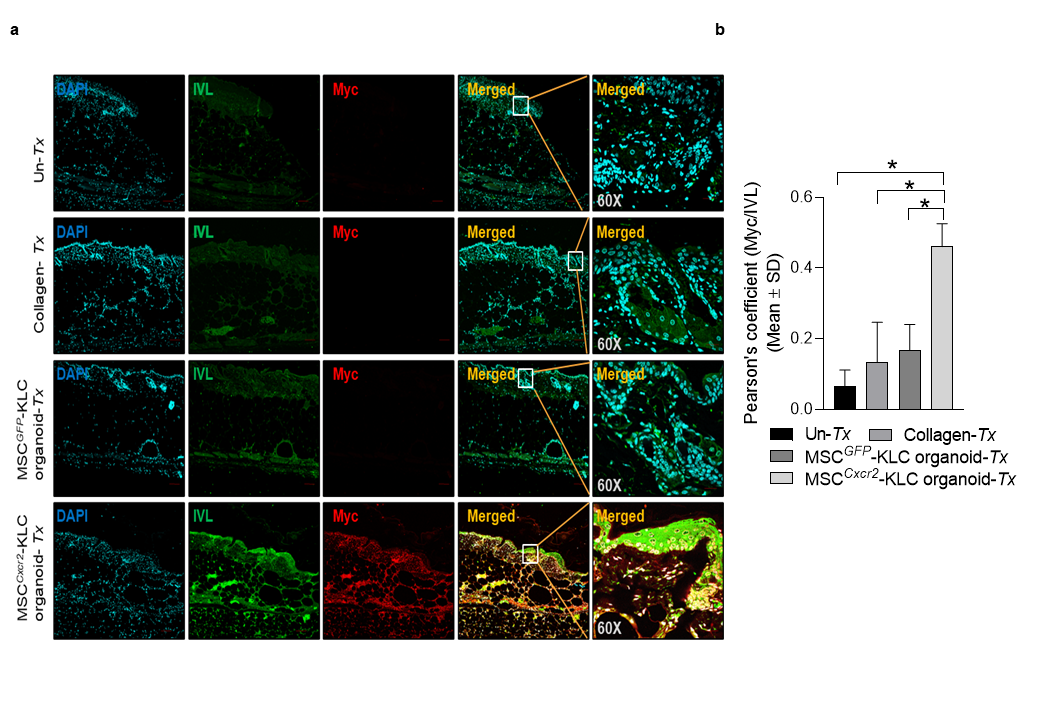
***Figure S12.*** ***Enhanced colocalization of Involucrin and Myc-tagged CXCR2 at regenerated wound tissue in type 2 diabetic wound grafted with bioengineered MSC-derived KLC organoids.*** (a) Representative confocal microscopy images of regenerated tissue sections immunostained with Involucrin and Myc tag depicting significantly higher (b) colocalization in the MSC*^Cxcr2^*-KLC organoid transplanted group. (n=3, *p<0.05 as compared with Un-*Tx* / MSC*^GFP^*-KLC organoid / Collagen implanted groups). (Scale bar: 10× - 100µm, 60× - 20µm).


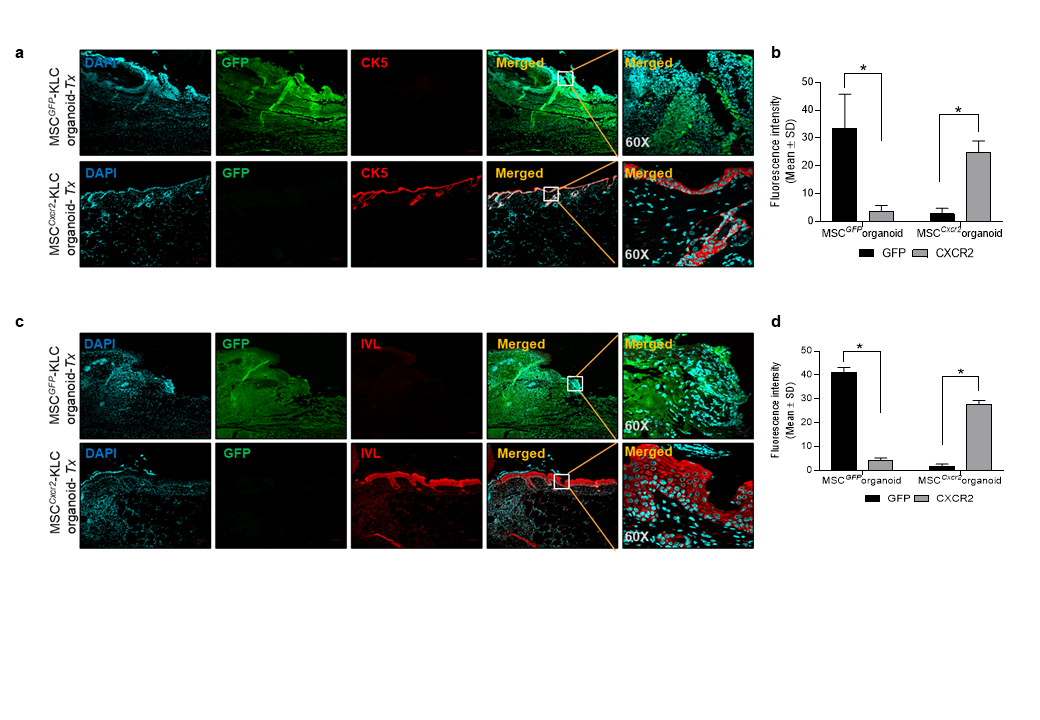
***Figure S13. Differential engraftment of bioengineered MSC-derived KLC organoids at type 2 diabetic wound.*** (a) Representative confocal microscopy images of regenerated tissue sections immunostained with GFP and CK5 revealed comparable fluorescence intensities of GFP and CK5. (b) Quantification of fluorescence intensities showed a higher GFP signal in the MSC*^GFP^*-KLC organoid and CK5 signal in the MSC*^Cxcr2^*-KLC organoid transplanted groups. Similarly, (c) representative confocal microscopy images regenerated skin sections immunostained with GFP and Involucrin (IVL) showing comparable fluorescence intensities of GFP and IVL. (d) Quantitation of the fluorescence intensities depicted higher GFP signal in the MSC*^GFP^*-KLC organoid and IVL signal in the MSC*^Cxcr2^*-KLC organoid transplanted groups. (n=3, *p<0.05 as compared with MSC*^GFP^*-KLC organoid grafted group). (Scale bar: 10× - 100µm, 60× - 20µm).


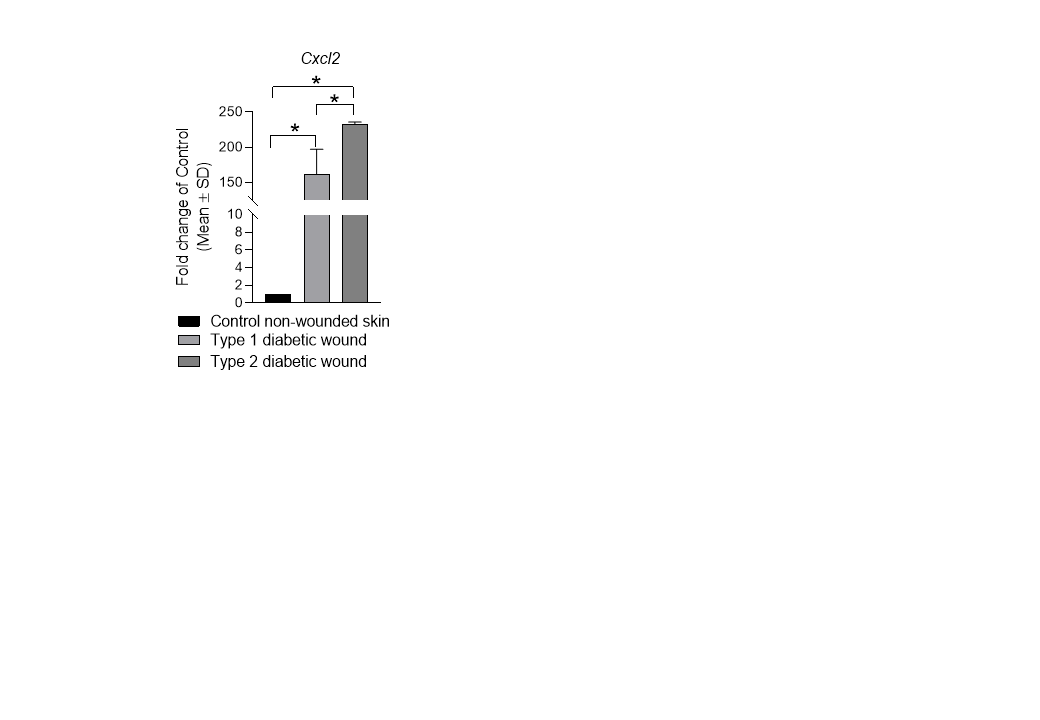


***Figure S14. CXCL2 expression at the wound sites of type 1 and type 2 diabetic wounds.*** Graph representing a significant increase in the expression of CXCL2 at type 1 and type 2 diabetic wound beds as compared to control non-wounded skin of non-diabetic mice. (n=3, *p<0.05 as compared with Control non-diabetic un-wounded skin tissue / Type 1 diabetic wound).
