# Supplementary figures and images for: Bioengineered MSCCxcr2 transdifferentiated keratinocyte-like cell-derived organoid potentiates skin regeneration through ERK1/2 and STAT3 signaling in diabetic wound
Source: Cell Mol Life Sci. 2024 Apr 10;81(1):172. doi: 10.1007/s00018-023-05057-3 (PMC11006766; doi:10.1007/s00018-023-05057-3)

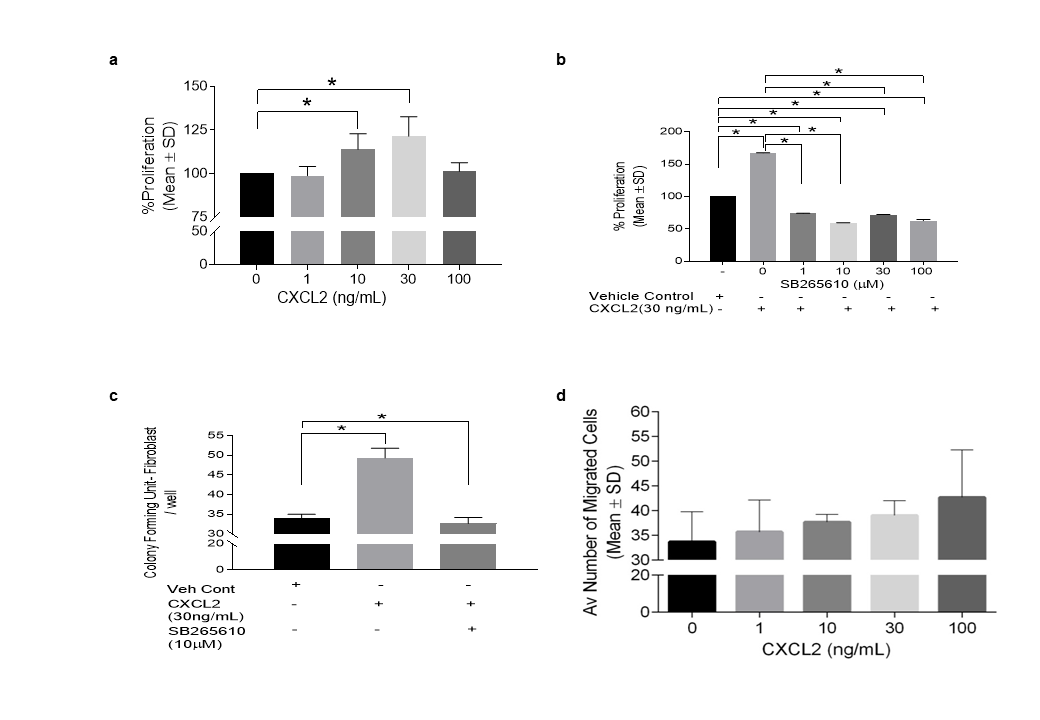

Supplement: Supplementary file 2 — Supplementary file2 (TIF 72 kb) [file 18_2023_5057_MOESM2_ESM.tif]

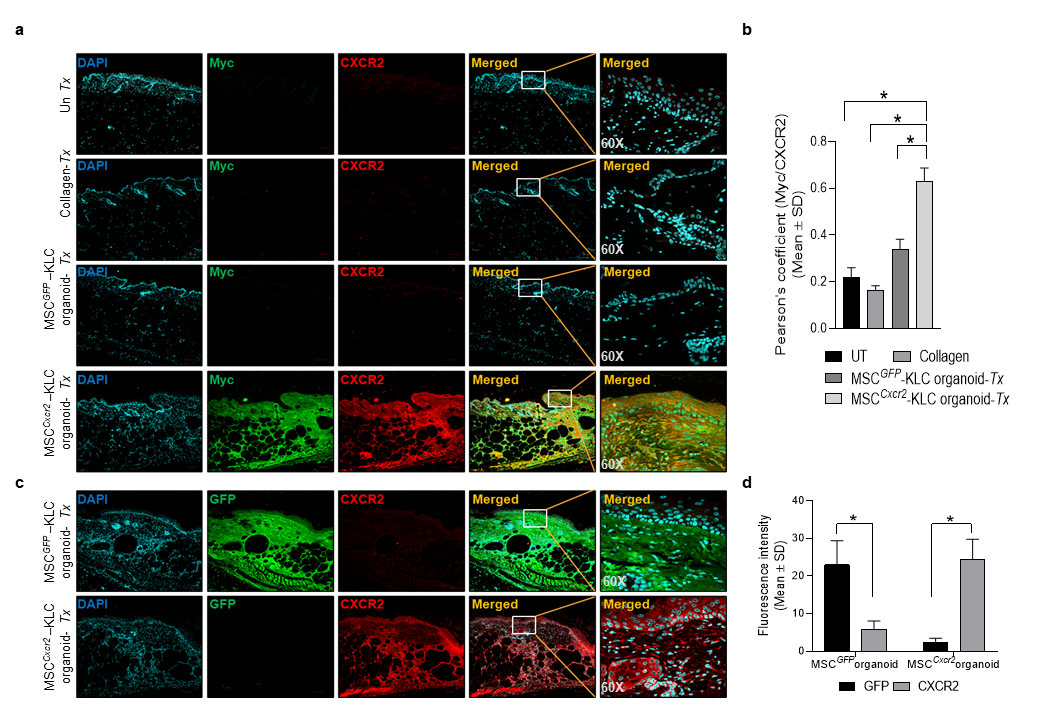

Supplement: Supplementary file 3 — Supplementary file3 (TIF 603 kb) [file 18_2023_5057_MOESM3_ESM.tif]

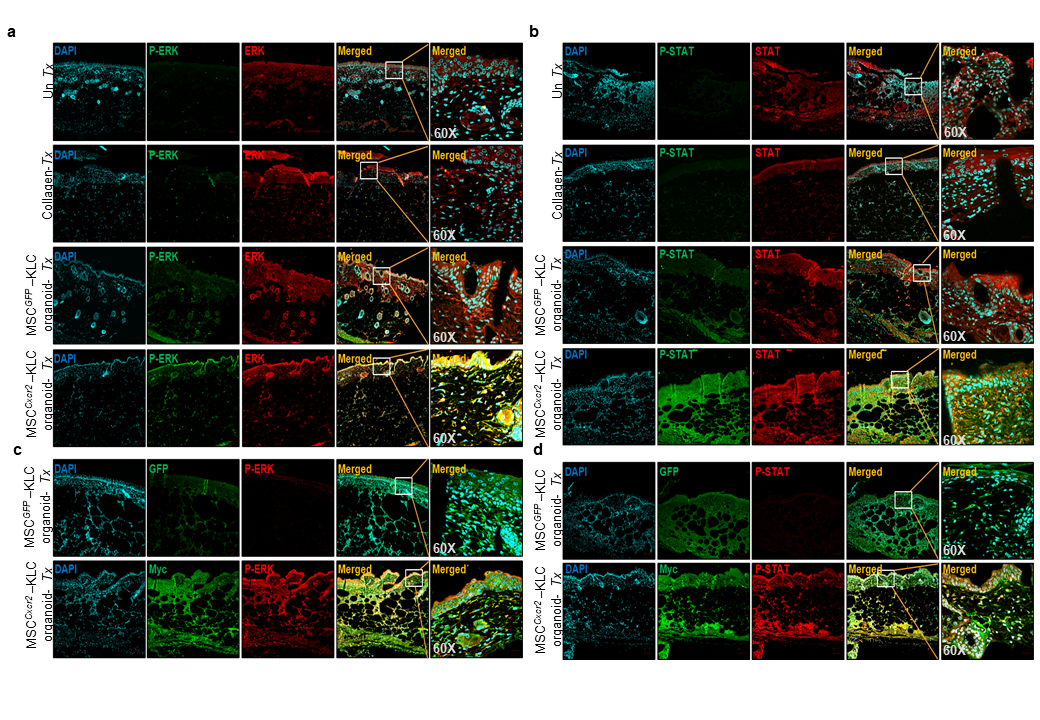

Supplement: Supplementary file 4 — Supplementary file4 (TIF 1049 kb) [file 18_2023_5057_MOESM4_ESM.tif]

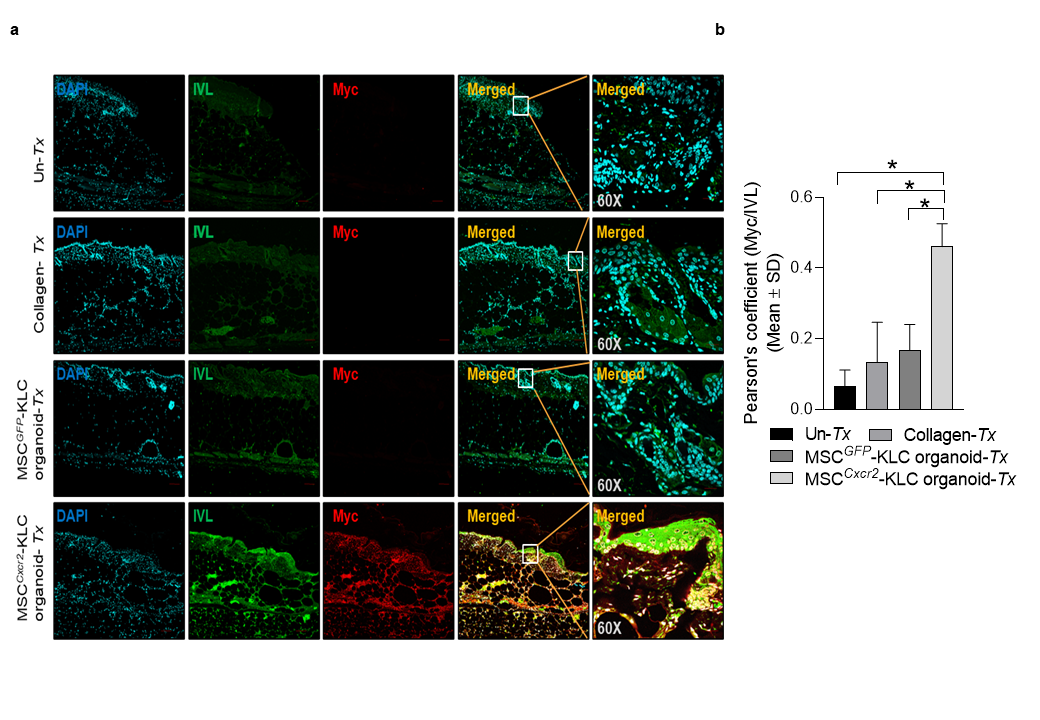

Supplement: Supplementary file 5 — Supplementary file5 (TIF 589 kb) [file 18_2023_5057_MOESM5_ESM.tif]

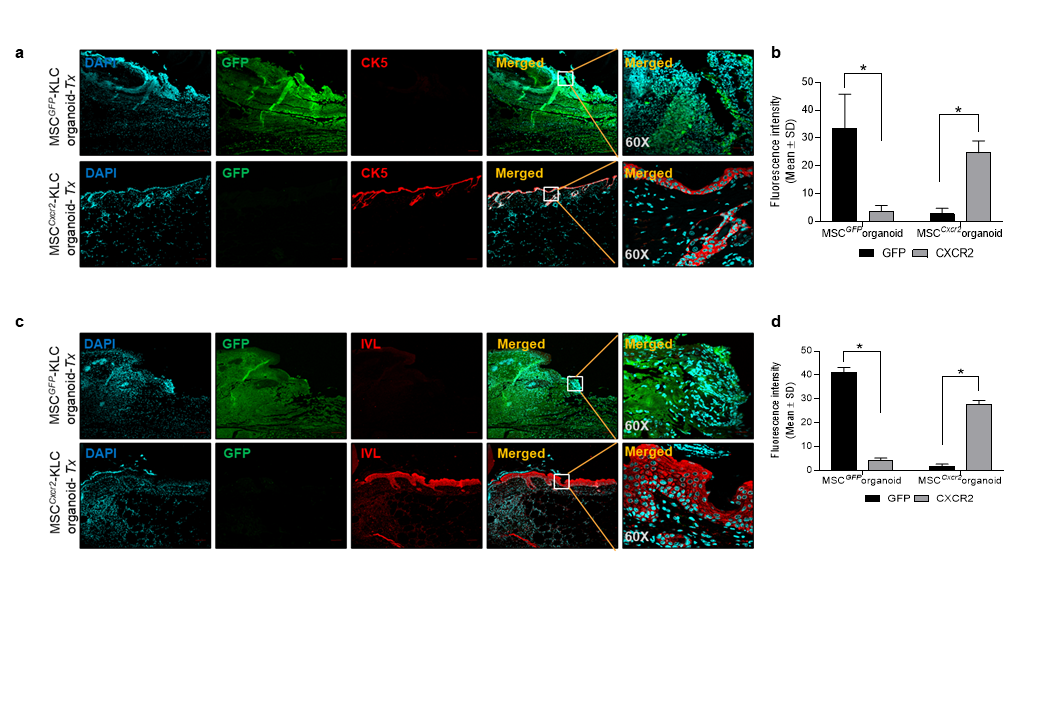

Supplement: Supplementary file 6 — Supplementary file6 (TIF 492 kb) [file 18_2023_5057_MOESM6_ESM.tif]

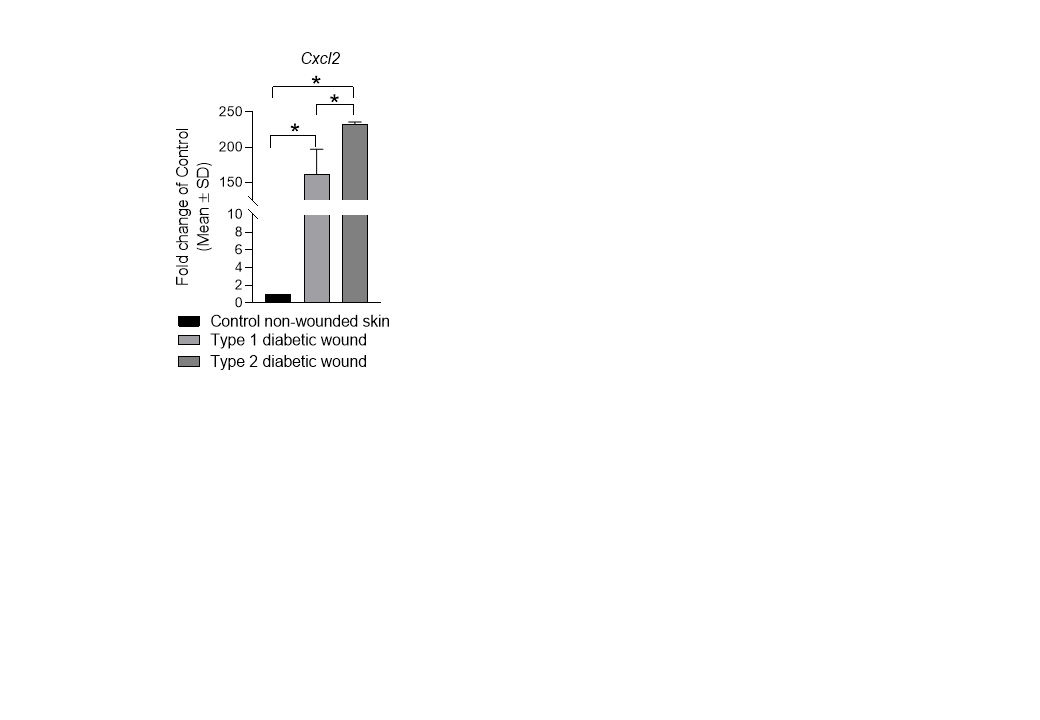

Supplement: Supplementary file 7 — Supplementary file7 (TIF 24 kb) [file 18_2023_5057_MOESM7_ESM.tif]

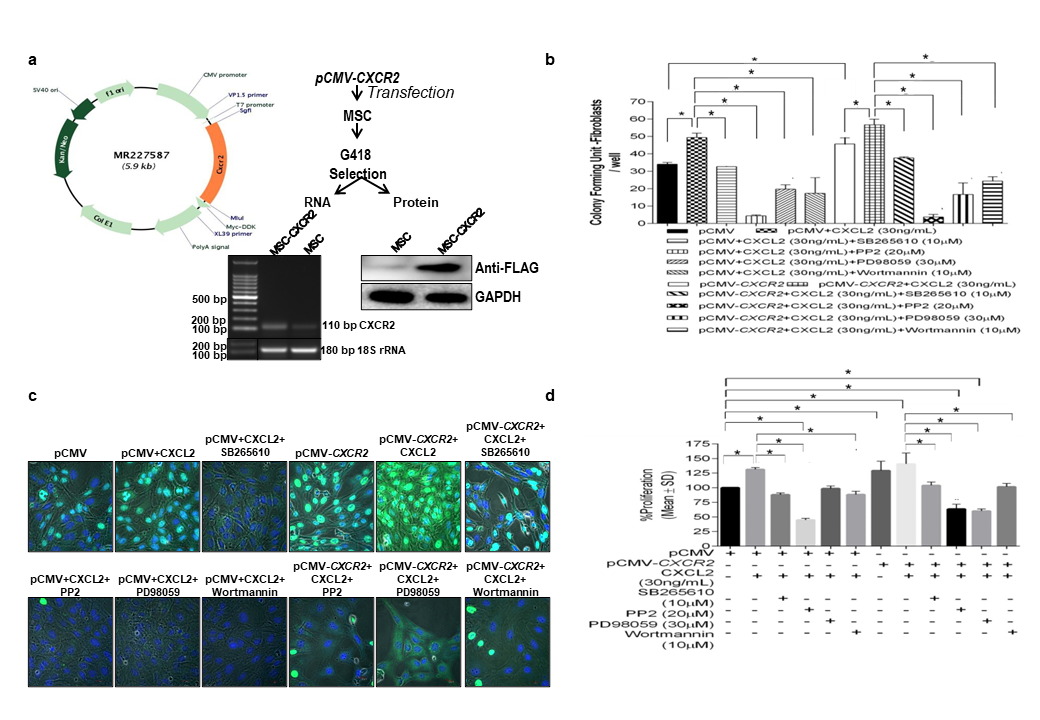

Supplement: Supplementary file 8 — Supplementary file8 (TIF 515 kb) [file 18_2023_5057_MOESM8_ESM.tif]

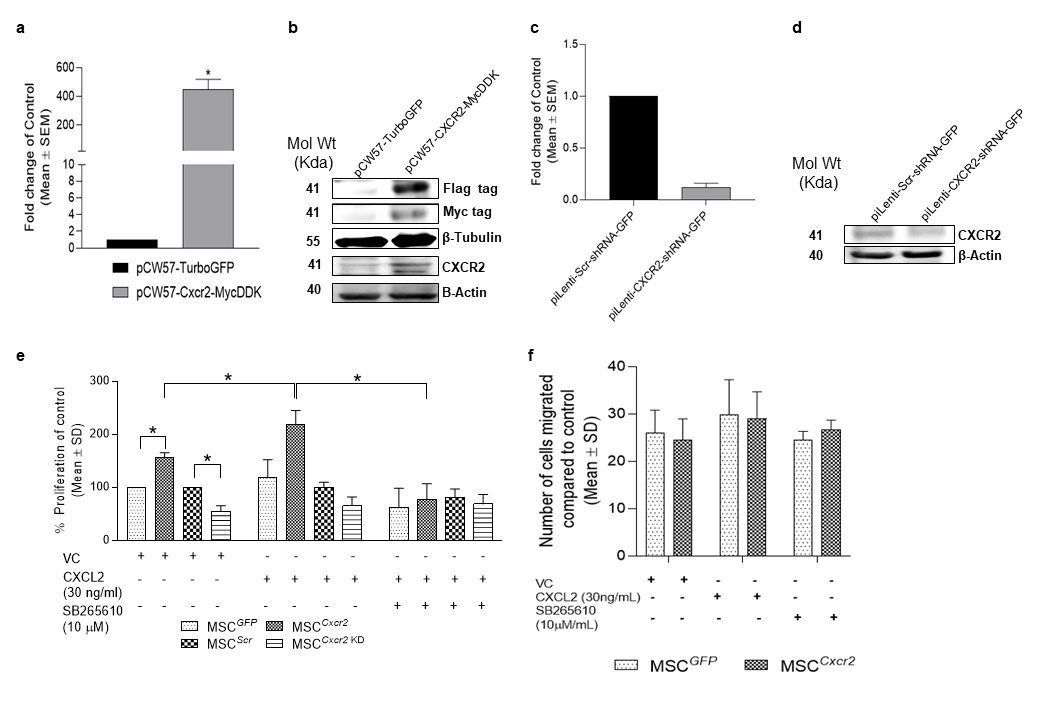

Supplement: Supplementary file 9 — Supplementary file9 (TIF 162 kb) [file 18_2023_5057_MOESM9_ESM.tif]

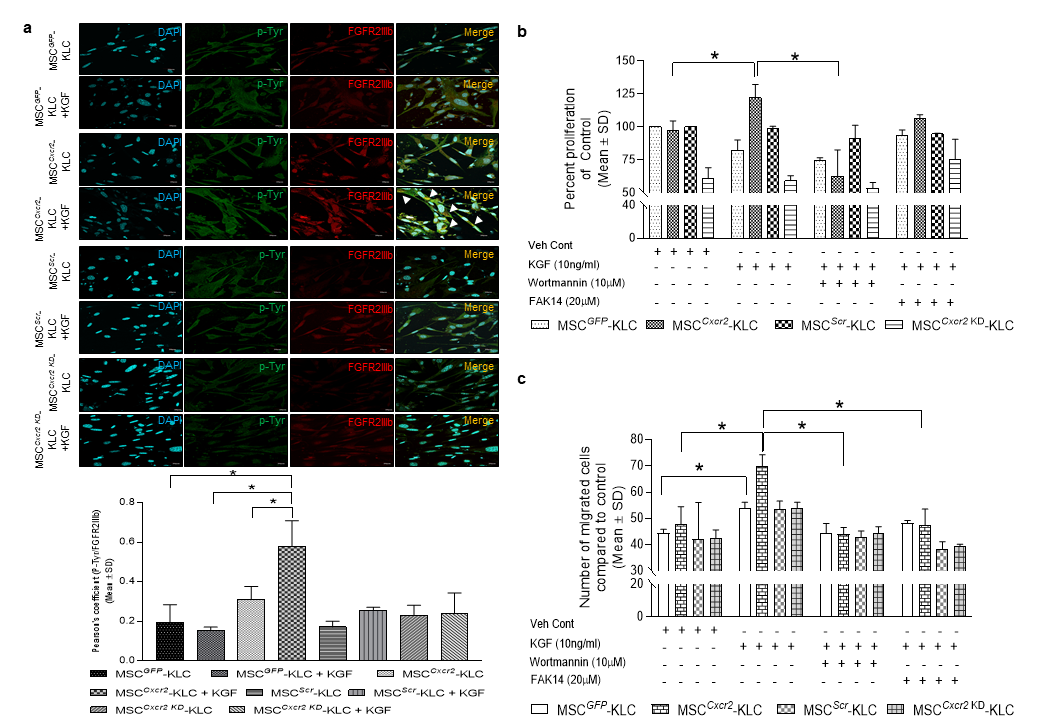

Supplement: Supplementary file 10 — Supplementary file10 (TIF 364 kb) [file 18_2023_5057_MOESM10_ESM.tif]

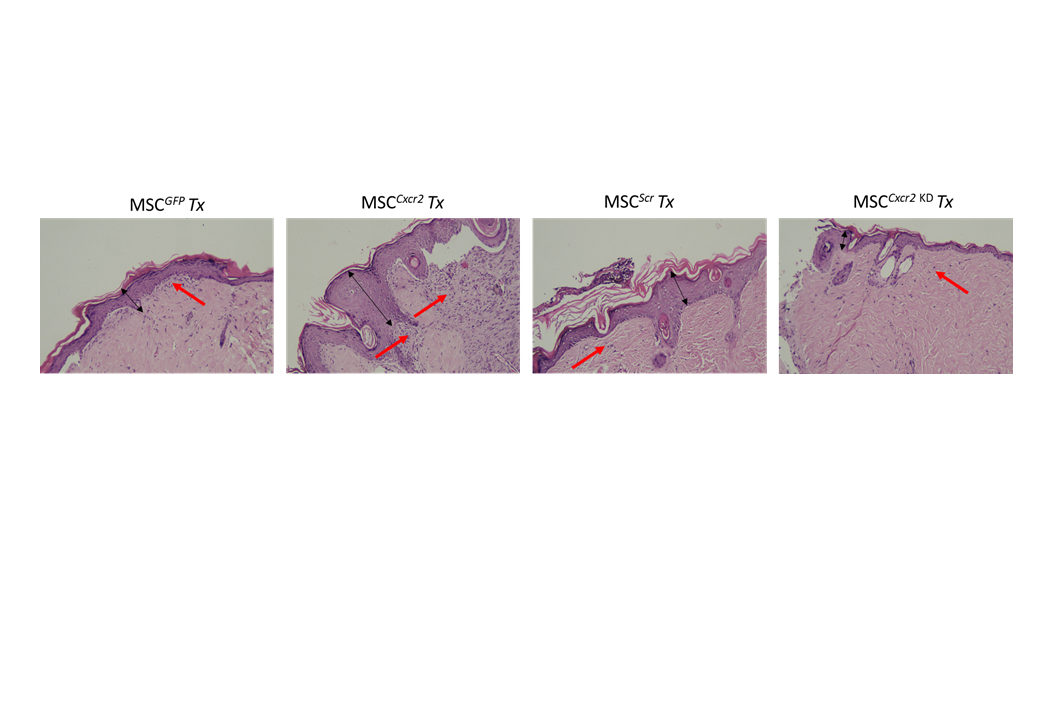

Supplement: Supplementary file 11 — Supplementary file11 (TIF 378 kb) [file 18_2023_5057_MOESM11_ESM.tif]

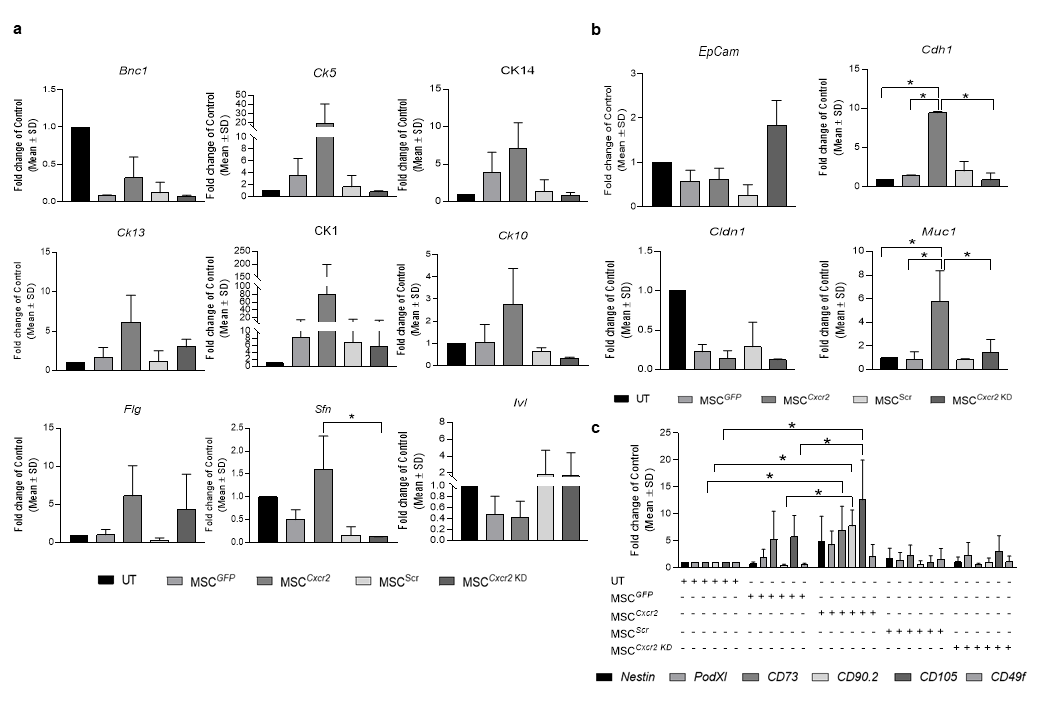

Supplement: Supplementary file 12 — Supplementary file12 (TIF 96 kb) [file 18_2023_5057_MOESM12_ESM.tif]

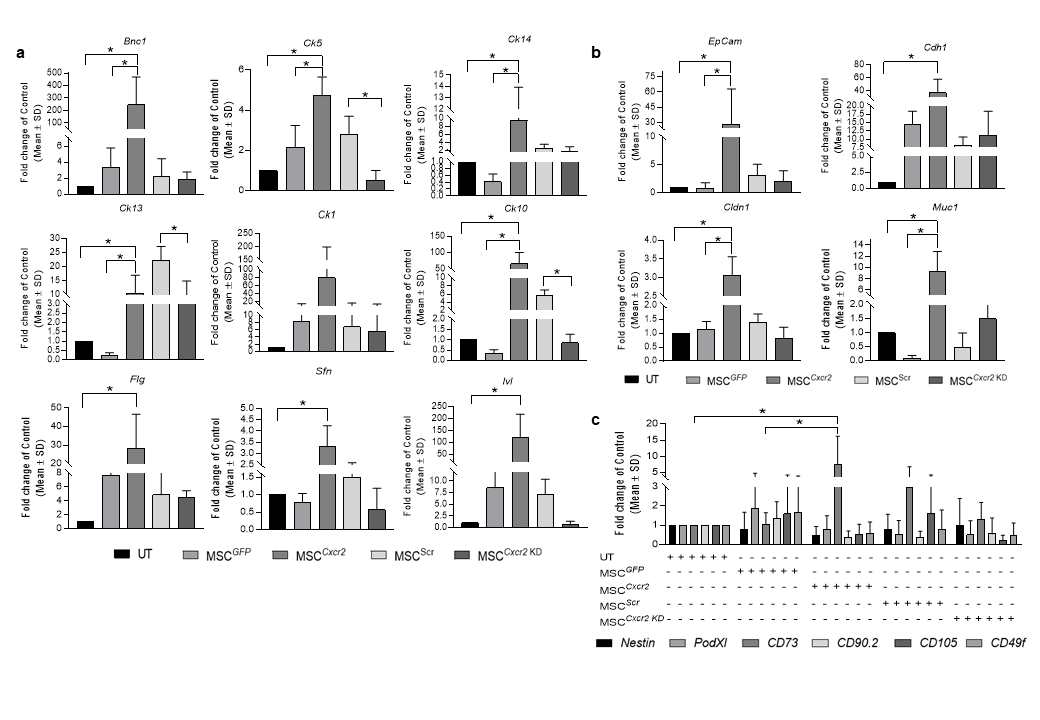

Supplement: Supplementary file 13 — Supplementary file13 (TIF 108 kb) [file 18_2023_5057_MOESM13_ESM.tif]

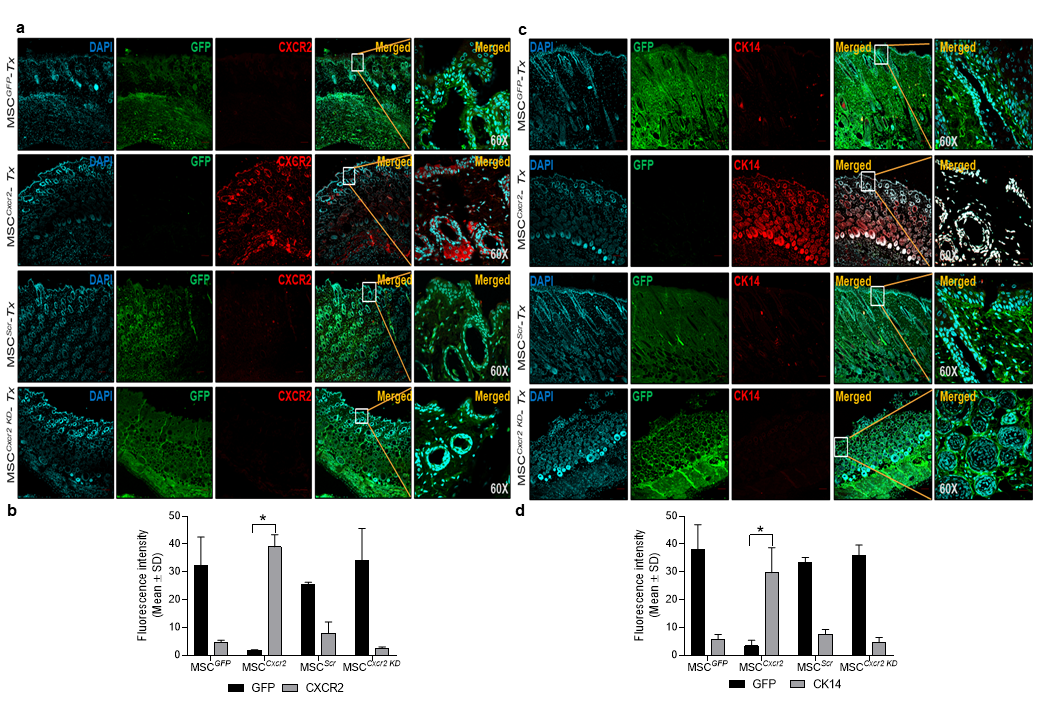

Supplement: Supplementary file 14 — Supplementary file14 (TIF 941 kb) [file 18_2023_5057_MOESM14_ESM.tif]

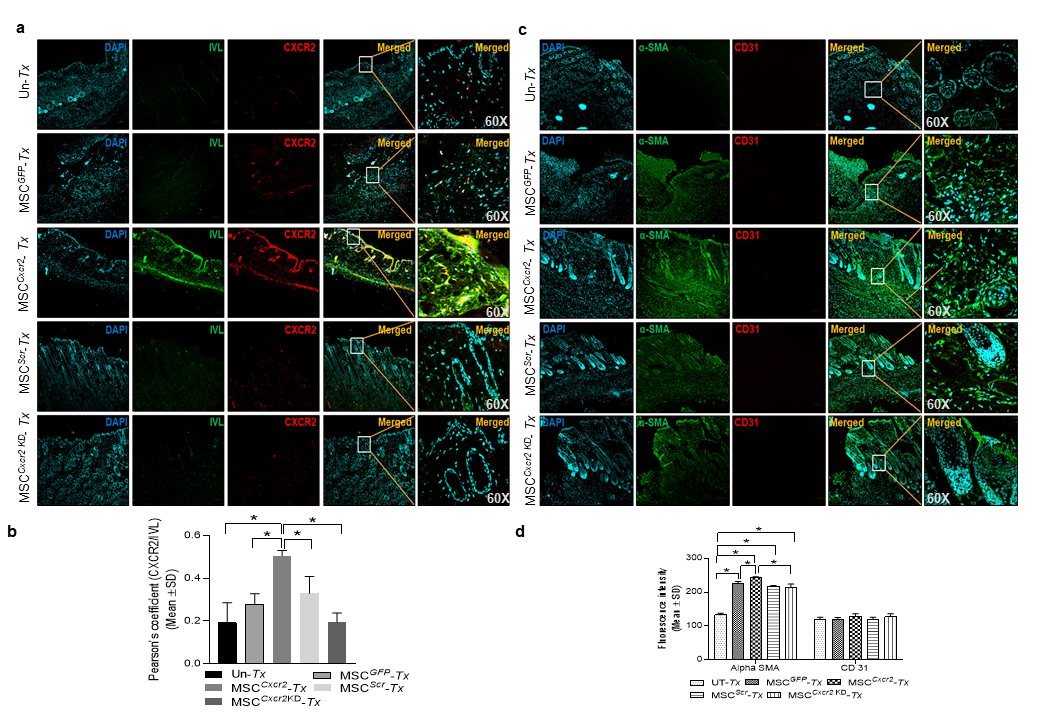

Supplement: Supplementary file 15 — Supplementary file15 (TIF 788 kb) [file 18_2023_5057_MOESM15_ESM.tif]
